# Dual-modality theranostics probes: sintegrative strategies for precision management of oncologic malignancies

**DOI:** 10.1093/lifemedi/lnaf037

**Published:** 2025-11-19

**Authors:** Xilin Jing, Yutao Li, Yijing Zhang, Yuqi Wang, Xiaohua Jia, Xing Yang, Kezhong Chen

**Affiliations:** Department of Thoracic Surgery, Peking University People’s Hospital, Beijing 100044, China; Thoracic Oncology Institute, Peking University People’s Hospital, Beijing 100044, China; Research Unit of Intelligence Diagnosis and Treatment in Early Non-small Cell Lung Cancer, Chinese Academy of Medical Sciences, Peking University People’s Hospital, Beijing 100044, China; Department of Thoracic Surgery, Peking University People’s Hospital, Beijing 100044, China; Thoracic Oncology Institute, Peking University People’s Hospital, Beijing 100044, China; Research Unit of Intelligence Diagnosis and Treatment in Early Non-small Cell Lung Cancer, Chinese Academy of Medical Sciences, Peking University People’s Hospital, Beijing 100044, China; Department of Nuclear Medicine, Peking University People’s Hospital, Beijing 100044, China; Department of Nuclear Medicine, Peking University People’s Hospital, Beijing 100044, China; Key Laboratory of Molecular Imaging of Chinese Academy of Sciences, Institute of Automation, Chinese Academy of Sciences, Beijing 100190, China; Department of Nuclear Medicine, Peking University People’s Hospital, Beijing 100044, China; Department of Thoracic Surgery, Peking University People’s Hospital, Beijing 100044, China; Thoracic Oncology Institute, Peking University People’s Hospital, Beijing 100044, China; Research Unit of Intelligence Diagnosis and Treatment in Early Non-small Cell Lung Cancer, Chinese Academy of Medical Sciences, Peking University People’s Hospital, Beijing 100044, China

**Keywords:** precision oncology, dual-modality probes, molecular imaging, theranostics

## Abstract

Cancer remains a formidable global public health challenge. Recent advancements in immunotherapy and targeted therapies have revolutionized diagnostic and therapeutic paradigms. Within this context, theranostics—an emerging field integrating molecular imaging with therapeutic interventions—has shown promise in achieving precision oncology. Central to theranostic platforms are dual-modality probes utilizing positron emission tomography, fluorescence, and magnetic resonance imaging technologies, which offer synergistic advantages such as complementary imaging modalities, intraoperative guidance, and real-time drug delivery monitoring. Despite growing research interest and early clinical trials, critical challenges persist in biosafety, metabolic stability, and imaging resolution. Structural optimization of probes and modality-specific selection based on cancer subtypes may address these limitations. This review systematically evaluates the design principles and clinical applications of dual-modality probes and proposes actionable strategies to enhance their translational potential.

## Introduction

Cancer incidence continues to rise globally. In 2020, more than 19 million new cancer cases and nearly 10 million cancer-related deaths were reported worldwide [1], an increase from 14.1 million new cases and 8.2 million deaths in 2012 [2]. By 2070, it is projected that over 34 million cancer cases will be diagnosed annually. Low-income countries are expected to be disproportionately affected, with cancer incidence in these regions predicted to increase by 400% over the next 50 years [3]. Precision diagnosis and treatment for different types of tumors remain critical challenges that the scientific community must address [4].

In recent years, molecular imaging has become a transformative tool in oncology [5]. It provides *in vivo* information about the expression levels and localization of specific biological markers offering direct insights into tumor behavior [6]. The main imaging modalities currently in use include positron emission tomography (PET), magnetic resonance imaging (MRI), fluorescence imaging (FLI), and photoacoustic imaging. Each modality has distinct advantages but also presents limitations [7]. For instance, FLI faces significant challenges in quantification, especially when imaging tissues deeper than a few millimeters. MRI, while providing excellent resolution, suffers from limited sensitivity, whereas PET, although highly sensitive, is constrained by relatively poor resolution [8]. Consequently, the integration of multiple imaging modalities through dual-modality molecular probes has emerged as a promising approach to overcoming these limitations and advancing imaging capabilities [9, 10].

Dual-modality molecular probes, which integrate two distinct imaging techniques into a single platform, represent a significant advancement in imaging technology. The development of dual-modality molecular imaging is crucial for the precise diagnosis and treatment of tumors. As cancer management increasingly shifts toward personalized and precision medicine, there is a growing demand for imaging modalities capable of not only detecting tumors but also assessing their molecular signatures and facilitating tailored treatments [11–13]. Traditional imaging methods may be limited by issues such as variable efficacy across different patients and insufficient specificity. In contrast, dual-modality probes offer enhanced targeting capabilities and enable individualized intraoperative navigation, thereby integrating diagnosis and treatment [14, 15]. For example, the combination of PET with near-infrared (NIR) fluorescence (NIRF), or MRI with NIRF, provides the unique ability to capture both functional and anatomical information simultaneously, facilitating precise tumor resection [16, 17]. Moreover, the molecular characteristics of the tumor can be better understood through the imaging results of dual-modality probes, enabling patient stratification to guide treatment decisions and predict prognostic outcomes [18].

**Figure 1.**
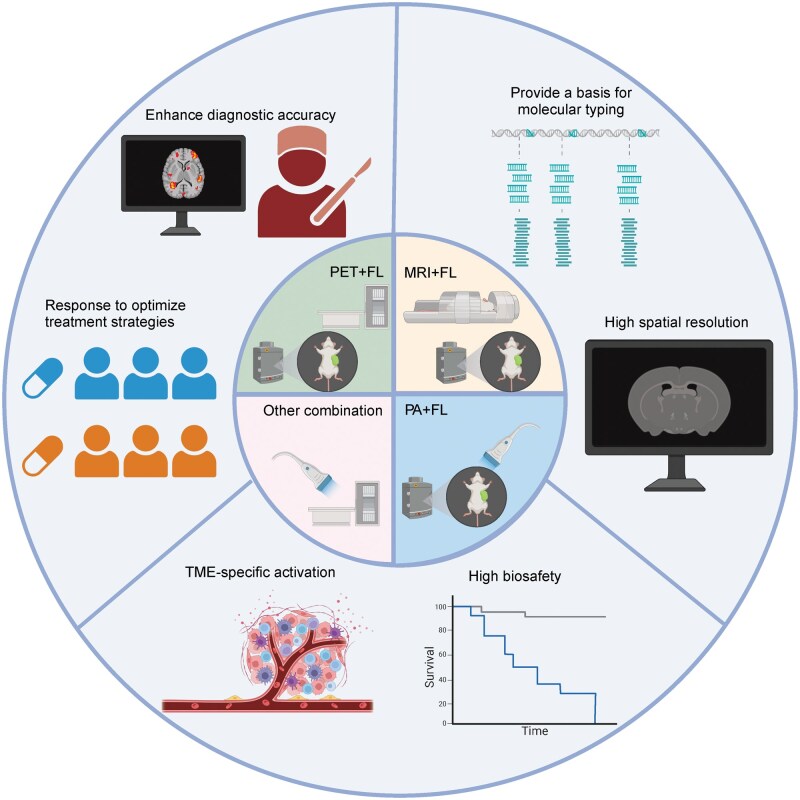
**Overview of dual-modality probe research.** Currently, dual-modality probes are primarily based on combinations of PET and FLI, MRI and fluorescence, as well as photoacoustic and fluorescence imaging. A variety of probes targeting different biomarkers are under preclinical investigation, while a limited number of PET-fluorescence dual-modality probes have progressed to clinical trials. The key advantages of dual-modality probes include: complementary imaging techniques that enhance diagnostic accuracy; the integration of diagnostic and therapeutic approaches for precise tumor treatment; real-time monitoring of drug responses to optimize therapeutic outcomes; and visualized molecular subtyping to advance precision oncology. PA, photoacoustic imaging; FL, fluorescence imaging. This figure was created with Biorender.

Over the past decade, substantial progress has been made in the development of dual-modality molecular probes, which are designed to enhance tumor detection accuracy, enable real-time monitoring of treatment responses, and guide therapeutic interventions with greater precision [19–21]. The performance of these probes has been further enhanced through the development and application of new materials and technologies, such as quantum dots (QDs), magnetic nanomaterials, and smart response probes [22–25]. This review examines the application and advancements of dual-modality molecular probes in cancer research, highlights the advantages of various dual-modality imaging combinations for precision tumor diagnosis and treatment, and discusses future directions and challenges in this field (Fig. 1).

## Structures of dual-modality molecular probes in cancers

Dual-modality molecular probes consist of several key structural components that work synergistically to enhance the precision and efficacy of both imaging and treatment. The structure of dual-modality probes is more complex than that of single-modality probes, as it facilitates multimodal integration. Generally, the core structure of dual-modality molecular probes includes four primary components: the targeting group, the reporter group, the linker, and the modification group.

### Targeting group

Dual-modality molecular probes primarily employ two types of targeting strategies: passive targeting and active targeting. Passive targeting exploits the enhanced permeability and retention effect, where nanoparticles or probes accumulate in tumor tissues due to the abnormal structure of tumor vasculature, such as hyperpermeable vasculatures and poor lymphatic drainage [26]. However, passive targeting may have problems such as low targeting ability, so many probes have introduced targeting groups for active targeting [27]. This group specifically binds to overexpressed receptors or antigens on the surface of tumor cells, allowing for selective accumulation at the tumor site. Active targeting enhances the specificity and sensitivity of the probe, improving both imaging and therapeutic outcomes.

**Figure 2.**
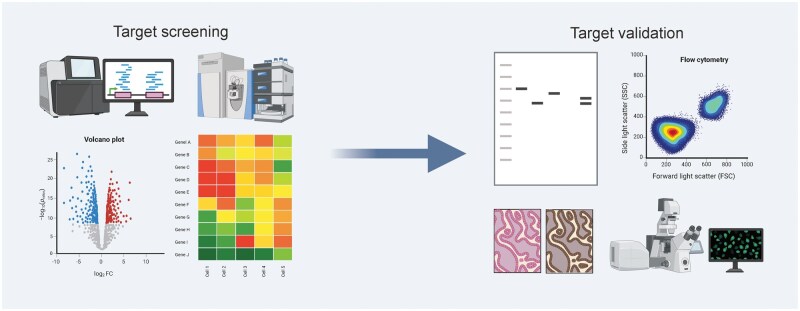
**From the screening of targets to their validation. **Probe targets are initially screened via multiomics approaches (e.g. transcriptomics and proteomics), followed by comprehensive validation using western blotting, flow cytometry, IHC, and confocal fluorescence microscopy to confirm their feasibility. This figure was created with BioRender.

Currently, target screening can be performed via multiomics approaches, followed by validation and evaluation through western blotting, immunohistochemistry (IHC), flow cytometry, confocal fluorescence microscopy, etc. (Fig. 2). After target identification, the targeting groups need to be designed, which mainly include monoclonal antibodies, nanoantibodies, peptide. Monoclonal antibodies have a high affinity for the target, but at the same time have high immunogenicity, which may trigger an immune response [28]. Peptides have small molecular weights and strong penetrability but may have insufficient affinity [29]. It is worth noting that nucleic acid aptamer, as an emerging targeting strategy, has attracted much attention in the field of precision cancer diagnosis and treatment in recent years. Aptamers are short, single-stranded nucleic acids that can fold into unique 3D structures, enabling them to bind selectively and with high affinity to specific tumor markers [30]. In dual-modality imaging, aptamers can be conjugated with different imaging reporter groups for use in techniques like fluorescence, PET, or MRI [31–33]. The advantages of aptamers include high specificity, flexible modifiability, low immunogenicity, and superior tissue penetration, making them an attractive alternative to traditional antibody-based targeting strategies for dual-modality probes.

### Reporter group

The reporter groups, serving as essential core components of dual-modality probes, comprise multiple elements that generate distinct signals detectable by imaging systems. These groups generate tumor-specific imaging signatures that drive precision diagnosis and therapy-guided decision-making in oncology. The integration of different imaging modalities requires distinct reporter groups tailored to each mode, such as radionuclides (e.g. ⁶⁴Cu, ¹⁸F, ⁶⁸Ga) for PET imaging, fluorescent dyes (e.g. ICG, IRDye800CW) for FLI, and contrast agents [e.g. Gd³^+^, superparamagnetic iron oxide nanoparticles (SPIONs)] for MRI [34, 35]. The utilization of dual reporters enables the simultaneous acquisition of complementary information by integrating the strengths of distinct imaging modalities.

### Linker

**Figure 3.**
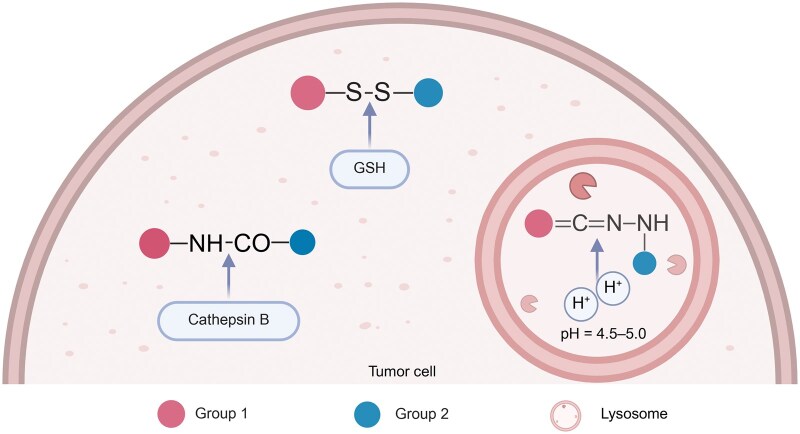
**Release mechanisms based on three representative “smart” linkers.** Several specialized chemical structures have been employed to design “smart” linkers that enable stimulus-responsive release. For instance, glutathione (GSH) can reduce disulfide bonds, the acidic microenvironment of lysosomes can hydrolyze hydrazone bonds, and tumor-overexpressed proteases (such as cathepsin B and MMPs) can cleave specific peptide bonds. These mechanisms facilitate the precise release of reporter groups or therapeutic agents in targeted tumor environments. This figure was created with BioRender.

The linker is a pivotal component of dual-modality molecular probes, serving to connect the targeting and reporter groups while maintaining their functional integrity. For PET-based dual-modality imaging, the chelator functions as a key linker by forming stable coordination complexes with radiometals. Examples include DOTA, NOTA, and DFO, which coordinate with radionuclides such as ^64^Cu, ^68^Ga, and ^89^Zr through nitrogen and oxygen donor atoms, ensuring *in vivo* stability and minimizing nonspecific release [16, 36, 37]. For some linkers, they may have multiple functions. Sun et al. [38] employed a diazole-based photo-click linker that not only covalently linked the targeting ligand and PET moiety via a cycloaddition reaction but also exhibited inherent fluorescence for optical imaging. Furthermore, “smart” linkers can be engineered to be stimuli-responsive, undergoing cleavage in response to tumor-specific conditions [39] (Fig. 3). For example, peptide linkers cleavable by matrix metalloproteinases (MMPs) overexpressed in the tumor microenvironment (TME) or acid-labile hydrazone bonds that hydrolyze in the acidic pH of tumor tissues can trigger the release of imaging agents or drugs in a site-specific manner [40–42]. Generally, different choices of linker significantly can influence the overall pharmacokinetics, bioavailability, and stability of the probe, as well as the efficiency of signal generation.

### Modification group

The modification group in dual-modality molecular probes plays a critical role in optimizing their pharmacokinetics, stability, and tumor-targeting efficiency. Polyethylene glycol (PEG) is a common modification group. Its introduction into the dual-modality probe can prolong the blood circulation time and improve the stability and water solubility of the probe [43, 44]. Su et al. [45] utilized SiO_2_ shells to reduce interference between MRI and fluorescence signals and enhance nanoparticle stability. The rich modification groups can meet people’s different needs for the performance of the probes and achieve personalized regulation, which is conducive to improving the performance of the dual-modality probes in the precision diagnosis and treatment of tumor patients.

In summary, the structure of dual-modality molecular probes represents a highly specialized and integrative design intended to enhance the precision of tumor diagnosis and treatment. These probes exhibit distinct variations in their compositional profiles, reflecting differences in design strategies and functional requirements. Each structural component plays a vital role in ensuring the probe effectively targets tumor cells, generates clear and accurate imaging signals, and maintains stability and functionality within the body.

## PET/fluorescence dual-modality molecular imaging

### Principles of PET/fluorescence dual-modality imaging

Positron emission tomography and FLI are two leading molecular imaging techniques that have significantly advanced cancer diagnosis and treatment monitoring. PET imaging involves the use of radiotracers to track metabolism *in vivo*, generating gamma photons through positron annihilation. These photons are captured by detectors and reconstructed to reflect tissue metabolism and function [46]. FLI, on the other hand, involves irradiating the sample with excitation light, causing fluorescent molecules to emit light after absorbing energy. The emitted fluorescence is captured by detectors, allowing for the generation of images that facilitate the observation and analysis of biomolecules or tissues [47].

Positron emission tomography/fluorescence dual-modality probes offer significant advantages by providing complementary information. While PET allows for whole-body imaging and the quantification of molecular processes, FLI delivers high-resolution, site-specific data that reveal intricate tumor details and facilitates precise excision [48, 49].

As the only type of dual-modality probe that has undergone human trials, combining both diagnostic and therapeutic properties, it is expected to be the first to be widely applied and to offer the greatest potential for translational value.

### Applications of PET/fluorescence dual-modality probes

#### Prostate-specific membrane antigen

Prostate-specific membrane antigen (PSMA) is highly expressed on the majority of prostate cancer (PCa) cells, making it a focal point of interest for imaging researchers [50]. As a prominent target, it demonstrates excellent efficacy in PCa imaging, and several PSMA ligands for PET imaging are now widely available globally [51]. In recent years, scientists have designed and developed a variety of PET/fluorescence dual-modality probes and carried out experimental verification *in vitro* and *in vivo*. The dual-modality probes targeting PSMA are helpful in achieving better results in the diagnosis and treatment of specific tumors.

Schottelius et al. [52] developed and evaluated a PSMA-targeted hybrid tracer, PSMA-I&F, which combines PET and FLI to enhance PCa surgery and diagnostics. Baranski et al. [53] synthesized a series of novel PSMA-targeting fluorescent dye conjugates of Glu-urea-Lys-HBED-CC and demonstrated that dual-labeled dye conjugates derived from PSMA-11 were effective for PSMA-specific detection of PCa lesions in the preoperative, intraoperative, and postoperative stages of PCa. To address one of the main challenges in achieving high contrast imaging at both early and late time points, Li et al. [54] optimized the pharmacokinetics of dual-modality probes based on oxalyldiaminopropionic acid-urea (ODAP-Urea) PSMA inhibitors. Using aptamer technology, Kong et al. [33] also developed dual-modality probes with good fluorescence properties and stability. Some new synthetic methods are gradually attracting the attention of scientists. d‘Orchymont et al. present the synthesis and characterization of bimodal rotaxane-based imaging agents, assembled using the cucurbit[6]uril (CB[6])-mediated alkyne-azide “click” reaction. They developed new routes and synthesized a PET/fluorescence dual-modality probe targeting PSMA, which is flexible and convenient [55].

Recent clinical trials have also demonstrated the feasibility of dual-modality probes targeting PSMA. Chen et al. [56] conducted the first in-human study of a novel PSMA-targeted dual-modal probe, 68Ga-P3, which integrates PET and FLI to precisely detect PCa and enable real-time surgical navigation, significantly improving surgical outcomes. Eder et al. present, for the first time, preoperative PET/CT imaging followed by fluorescence-guided surgery using a PSMA-11-derived peptidomimetic PSMA-targeting hybrid molecule. By injecting ^68^Ga-Glu-urea-Lys-(HE)3-HBED-CC-IRDye800CW (^68^Ga-PSMA-914), they performed PET imaging and fluorescence-guided DaVinci-assisted radical prostatectomy in a patient with PCa and showed promising results [57]. Fu et al. [27] constructed a PET/fluorescence probe [^68^Ga]Ga-NYM016 which was evaluated in patients with PCa and prostatitis demonstrating its good clinical application prospect. It is worth mentioning that PET/fluorescence dual-modality probes can be combined with photodynamic therapy (PDT) to further realize the integration and personalization of tumor diagnosis and treatment. Harmatys et al. successfully constructed ^64^Cu-LC-Pyro targeting PSMA, which enables effective single-dose tumor ablation by PDT while achieving dual-modality imaging. The peptide adaptor embedded in the structure effectively prolongs the plasma circulation time, and its combination with dual-modality imaging technology is conducive to accurate diagnosis and image-guided surgical resection [58]. PET/fluorescence dual-modality probes targeting PSMA hold significant promise for the precision diagnosis and treatment of PCa. Their effectiveness has been validated in clinical trials, although further large-scale experimental validation is required.

#### CD105 and human epidermal growth factor receptor 2

CD105 and human epidermal growth factor receptor 2 (HER2) have been shown to be frequently highly expressed in breast cancer [59, 60]. Therefore, scientists have developed a variety of PET/fluorescence dual-modality probes targeting CD105 or HER2. Zhang et al. [61, 62] developed a TRC105-based dual-labeled molecular imaging probe (⁶⁴Cu-NOTA-TRC105-800CW) to detect the expression of tumor angiogenesis marker CD105 in both breast cancer models and lung metastasis models of breast cancer by PET and NIRF imaging. It provides potential application value for clinical diagnosis and image-guided surgery. Similarly, Hong et al. [63] constructed ^89^Zr-Df-TRC105-800CW probe, which also confirmed its excellent specificity in the lung metastasis model of breast cancer.

Hollow mesoporous silica nanoparticles (HMSNs) have recently attracted growing interest due to their significant potential as an appealing nanoplatform for cancer imaging and therapy. Chen et al. developed a HMSN for active tumor targeting that enabled dual-modality PET and NIRF imaging and significantly improved drug delivery efficiency. By targeting the tumor angiogenesis marker CD105, HMSN showed a tumor uptake of up to 10%ID/g *in vivo*, more than three times higher than that of the untargeted group, and its anticancer drug loading capacity was 3–15 times higher than that of conventional mesoporous silica nanoparticles, providing a new nanoplatform for accurate cancer diagnosis and treatment [64].

Multifunctional zinc oxide (ZnO) nanoparticles have garnered increasing research interest due to their excellent multimodal imaging capabilities. Hong et al. [65] developed novel red fluorescent ZnO nanoparticles and conjugated them with ^64^Cu (*t*₁/₂ = 12.7 h) and TRC105 to construct dual-modality probes targeting CD105, making them a promising candidate for cancer theranostics.

HER2 as an effective target has also attracted the attention of scientists in the design of dual-modality probes. Sampath et al. synthesized (^64^Cu-DOTA)_*n*_-trastuzumab-(IRDye800)_*m*_ for detection of primary tumors and metastases of HER2-positive breast cancer. The results showed that this imaging agent was effective in detecting metastases in the lung and other sites in a mouse model, which was not achieved with conventional ¹⁸F-FDG-PET imaging, indicating its potential application in the staging and intraoperative resection of HER2-positive breast cancer [66]. Adumeau et al. developed a site-specific conjugation strategy based on a trivalent platform for the synthesis of dual-labeled immunoconjugates of HER2-positive tumors for dual-modality imaging with PET and NIRF. This platform was specifically coupled to trastuzumab by glycosyl engineering. The strategy outperforms current clinical standards in reducing heterogeneity, improving fluorescence stability, and reducing nonspecific uptake in spleen and liver [37]. Significantly reduced photon scattering and minimal tissue autofluorescence in the second biological transparency window (NIR-II; 1000–1700 nm) enhance the resolution of *in vivo* biological imaging compared to traditional NIR fluorophores (about 700–900 nm) [67]. Cao et al. [68] developed a novel HER2-targeting peptide probe (DOTA-ZC02-ICG) for NIR-II FLI and PET dual-modality tumor imaging, which showed excellent tumor targeting performance and imaging effect. The development of dual-modality probes targeting HER2 has contributed to better diagnosis and treatment of HER2-positive tumors.

The success of dual-modality probes targeting HER2 and PSMA suggests the necessity of identifying specific antigens for particular cancer types. These biomarkers should exhibit stable expression in tumor tissues while showing a significant differential expression when compared to normal tissues. With the advancement of multiomics technologies, integrating molecular subtyping for different cancers will be a key direction for the future development of dual-modality probes.

#### Other targets

Due to the high heterogeneity and complexity of tumors, a variety of PET/fluorescence dual-modality molecular probes have been developed to target distinct tumor groups. Kimura et al. [69] report the development of a dual-labeled knottin peptide targeting α_v_β_3_ and α_v_β_5_ integrins and conjugated it to both NIRF and PET imaging agents. Diao et al. [70] synthesized a dual-modality probe targeting α_v_β_3_ integrin based on the bioorthogonal chemistry of vinyl tetrazolium. Aiming at the same target, Sun et al. combined NIR-II with PET to develop a novel probe ^68^Ga-CHS2 for precise imaging and intraoperative navigation of tumors. The probe is highly synthesized by a base-catalyzed thiol-yne reaction, which achieves good tumor targeting, high-contrast imaging, and tumor resection guidance in mouse models, showing the potential for clinical translation [71].

Gastrin-releasing peptide receptor (GRPR) is highly expressed in a variety of cancers and has also become an important target group for dual-modality probes [72, 73]. Related clinical trials have also been carried out. Li et al. developed a novel ^68^Ga-IRDye800CW-BBN PET/NIRF dual-modality imaging probe targeting GRPR, which was studied in 14 glioblastoma multiforme patients. The results revealed a strong correlation between preoperative positive PET uptake and intraoperative NIRF signals [74]. Chen et al. [16] demonstrated the feasibility of integrated preoperative and intraoperative targeted imaging through the same molecular receptor by performing preoperative dual-modality imaging evaluation as well as intraoperative fluorescein-guided surgery in 10 glioma patients.

ETA receptor, EphB4, folate receptor, PARP1, UPAR, and so on as the targets of PET/fluorescence dual-modality probes have good research prospects in the diagnosis and treatment of glioblastoma [38, 75–78] (Table 1). As a highly expressed protein in tumor-associated fibroblasts, FAP has attracted much attention from scientists in the dual-modality diagnosis and treatment of a variety of cancers [79, 80]. It is worth mentioning that Kong et al. designed the [^68^Ga]Ga-NOTA-Cy5-R1 probe targeting PD-L1. By combining PET and FLI, the noninvasive detection of tumor PD-L1 expression can be realized, and the change of PD-L1 expression can be monitored during immunotherapy, which provides an important tool for the precise application of immunotherapy [32]. With the deepening of the research on tumor mechanisms and key molecules, scientists have developed and designed dual-modality molecules for different targets, which helps to improve the specificity of imaging for different cancers and realize the personalized diagnosis and treatment of patients [81–95].

**Table 1.** Summary of key information on PET/fluorescence dual-modality probes in oncology

Author	Year	Type of research	Type of cancer	Imaging technologies	Radionuclide	Linker	Dye	Targeting group	Receptor	Reference
**Chen, Zhang et al.**	2024	Clinical	Glioma	PET/NIR-I	^68^Ga	NOTA	IRDye800CW	Bombesin	GRP receptor	[16]
**Chen, Xu et al.**	2025	Clinical	Prostate cancer	PET/NIR-I	^68^Ga	NA	Indocyanine green analog	ODAP-Urea	PSMA	[56]
**Eder, Omrane et al.**	2021	Clinical	Prostate cancer	PET/NIR-I	^68^Ga	HBED-CC	IRDye800CW	Glu-urea-Lys-(HE)_3_	PSMA	[57]
**Fu, Lou et al.**	2024	Clinical	Prostate cancer	PET/NIR-I	^68^Ga	NOTA	Cy7	NYM016	PSMA	[27]
**Li, Zhang et al.**	2018	Clinical	Glioblastoma	PET/NIR-I	^68^Ga	NOTA	IRDye800CW	Bombesin	GRP receptor	[74]
**Adumeau, Raavé et al.**	2022	Preclinical	Ovarian cancer	PET/NIR-I	^89^Zr	DFO	IRDye800CW	Trastuzumab	HER2	[37]
**An, Kommidi et al.**	2017	Preclinical	Lung cancer	PET/NIR-I	^18^F	Boronate	Pentamethine cyanine	Passive targeting	Passive targeting	[101]
**Ariztia, Jouad et al.**	2022	Preclinical	Glioblastoma	PET/NIR-I	^18^F	N/A	Cy5	c(RGDfK)	Integrin α_v_β_3_	[106]
**Baranski, Schäfer et al.**	2018	Preclinical	Prostate cancer	PET/NIR-I	^68^Ga	HBED-CC	AlexaFluor488, DyLight800, fluorescein isothiocyanate, IRDye800CW	Glu-urea-Lys	PSMA	[53]
**Cai, Chen et al.**	2007	Preclinical	Glioblastoma	PET/NIR-I	^64^Cu	DOTA	QD705	c(RGDfK)	Integrin α_v_β_3_	[97]
**Cao, Li et al.**	2023	Preclinical	Breast cancer and ovarian cancer	PET/NIR-II	^68^Ga	DOTA	ICG	ZC01, KSP, ZC02	HER2	[68]
**Carlucci, Carney et al.**	2015	Preclinical	Glioblastoma	PET/fluorescence	^18^F	N/A	BODIPY	2H-phthalazin-1-one	PARP1	[77]
**Chen, Hong et al.**	2014	Preclinical	Breast cancer	PET/NIR-I	^64^Cu	NOTA	ZW800	TRC105	CD105	[64]
**Chen, Li et al.**	2008	Preclinical	Glioblastoma	PET/NIR-I	^64^Cu	DOTA	QD705	VEGF	VEGFR	[98]
**Cheng, Kamkaew et al.**	2016	Preclinical	Breast cancer	PET/fluorescence	^64^Cu	Ce6	Ce6	Passive targeting	Passive targeting	[43]
**Deng, Wang et al.**	2015	Preclinical	Colon adenocarcinoma	PET/NIR-I	^64^Cu	DOTA	Cy5.5	Neurotensin analogue	Neurotensin receptor	[91]
**Diao, Lu et al.**	2025	Preclinical	Ovarian cancer	PET/NIR-I	^18^F	Vinyltetrazine	Cy5	P12d	Integrin α_v_β_3_	[70]
**d‘Orchymont, Holland**	2023	Preclinical	Prostate cancer	PET/fluorescence	^68^Ga	DFO	Fluorescein	Lys-urea-Glu	PSMA	[55]
**Gariglio, Bendova et al.**	2024	Preclinical	Squamous cell carcinoma	PET/NIR-I	^68^Ga	TRAP, FSC	Sulfo-Cy5.5	Trp-(N-Me)Nle-Asp-1-Nal-NH2	Cholecystokinin-2 receptor	[84]
**Ghosh, Hernandez et al.**	2017	Preclinical	Colorectal cancer	PET/NIR-I	^68^Ga	DO2A	IRDye800	TOC	Somatostatin receptor	[95]
**Ghosh, Rodriguez et al.**	2017	Preclinical	Pancreatic cancer	PET/NIR-I	^64^Cu	DO2A	IRDye800CW	TOC	Somatostatin receptor	[94]
**Hall, Pinkston et al.**	2012	Preclinical	Prostate cancer	PET/NIR-I	^64^Cu	DOTA	IRDye800CW	mAb7, mAb153	EpCAM	[86]
**Harmatys, Overchuk et al.**	2018	Preclinical	Prostate cancer	PET/fluorescence	^64^Cu	Porphyrin	Pyropheophorbide a	Urea-based small-molecule PSMA targeting ligand	PSMA	[58]
**Harmsen, Medine et al.**	2021	Preclinical	Ovarian cancer	PET/NIR-I	^89^Zr	N/A	Silica nanoparticle	CAR-T	hCEA	[108]
**Hautiere, Vivier et al.**	2024	Preclinical	Glioblastoma	PET/NIR-I	^89^Zr	DFO	IRDye800CW	axiRA63	ETAreceptor	[75]
**Hernandez, Sun et al.**	2016	Preclinical	Hepatocellular cancer	PET/NIR-I	^89^Zr	DFO	ZW800	YY146	CD146	[82]
**Hong, Wang et al.**	2015	Preclinical	Breast cancer	PET/Fluorescence	^64^Cu	NOTA	ZnO nanoparticles	TRC105	CD105	[65]
**Hong, Zhang et al.**	2012	Preclinical	Breast cancer experimental lung metastasis	PET/NIR-I	^89^Zr	Df-Bz-NCS	IRDye800CW	TRC105	CD105	[63]
**Houghton, Zeglis et al.**	2015	Preclinical	Pancreatic cancer	PET/NIR-I	^89^Zr	DFO	DIBO-FL	5B1	CA19.9	[81]
**Hu, Huang et al.**	2014	Preclinical	Glioblastoma	PET/NIR-I	^64^Cu	N/A	AuNCs	Passive targeting	Passive targeting	[104]
**Hu, Wang et al.**	2015	Preclinical	Prostate cancer	PET/NIR-I	^18^F	Fluoropropionyl	QD705	b-Glu-RGD-BBN	Integrin α_v_β_3_, GRPR	[100]
**Huang, Xiong et al.**	2014	Preclinical	Glioblastoma	PET/NIR-I	^64^Cu	DOTA	Cy5.5	TNYL-RAW	EphB4	[76]
**Kasten, Jiang et al.**	2020	Preclinical	Glioma	PET/NIR-I	^64^Cu, ^68^Ga	NOTA	IRDye800	MMP-14-binding peptide or substrate-binding peptide	MMP-14	[89]
**Kimura, Miao et al.**	2010	Preclinical	Glioblastoma and melanoma	PET/NIR-I	^64^Cu	DOTA	Cy5.5	Knottin peptide	Integrin α_v_β_3_ and α_v_β_5_	[69]
**Kong, Liu et al.**	2024	Preclinical	Melanoma	PET/NIR-I	^68^Ga	NOTA	Cy5	R1	PD-L1	[32]
**Kong, Peng et al.**	2024	Preclinical	Prostate cancer	PET/NIR-I	^68^Ga	NOTA	Cy5	Aptamer	PSMA	[33]
**Li, Duan et al.**	2022	Preclinical	Prostate cancer	PET/NIR-I	^68^Ga	DOTA	ICG analogue	ODAP-Urea	PSMA	[54]
**Li, Li et al.**	2023	Preclinical	Head and neck cancer	PET/NIR-I	^68^Ga	DOTA	ICG	FAP-2286	FAP	[79]
**Li, Wei et al.**	2021	Preclinical	Pancreatic cancer	PET/NIR-I	^89^Zr	DFO	IRDye800CW	ICAM-1 mAb	ICAM-1	[87]
**Lu, Fu et al.**	2024	Preclinical	Hepatocellular cancer	PET/NIR-I	^18^F	N/A	BODIPY	Lactobionic acid derivative	lasialoglycoprotein receptor	[88]
**Lu, Pham et al.**	2018	Preclinical	Colorectal cancer	PET/fluorescence	^124^I	N/A	Fluorescein	A5B7	CEA	[83]
**Luo, England et al.**	2017	Preclinical	Pancreatic cancer	PET/NIR-I	^64^Cu	NOTA	ZW800	Heterodimer	CD105, TF	[99]
**Lwin, Minnix et al.**	2023	Preclinical	Colorectal cancer	PET/NIR-I	^89^Zr	DFO	IRDye800	M5A	CEA	[44]
**Pang, Chen et al.**	2019	Preclinical	Glioblastoma	PET/fluorescence	^68^Ga	DOTA	Green fluorescent protein	Passive targeting	Passive targeting	[109]
**Paulus, Desai et al.**	2015	Preclinical	Prostate cancer	PET/NIR-I	^18^F	N/A	BODIPY	Bombesin analog	GRP receptor	[72]
**Pérez-Medina, Abdel-Atti et al.**	2014	Preclinical	Breast cancer	PET/NIR-I	^89^Zr	DFO	DiIC12 (5)-DS	Passive targeting	Passive targeting	[102]
**Pringle, Chan et al.**	2022	Preclinical	Chondrosarcoma	PET/NIR-I	^89^Zr	DFO	IRDye800CW	Anti-MT1-MMP	MT1-MMP	[90]
**Rodriguez, Wang et al.**	2016	Preclinical	Lung cancer and prostate cancer	PET/NIR-I	^18^F	DBO	Heptamethine cyanine	Cetuximab, anti-EpCAM mAb	EpCAM, EGFR	[103]
**Sampath, Kwon et al.**	2010	Preclinical	Breast cancer	PET/NIR-I	^64^Cu	DOTA	IRDye800	Trastuzumab	HER2	[66]
**Schottelius, Wurzer et al.**	2019	Preclinical	Prostate cancer	PET/fluorescence	^68^Ga, ^177^Lu	DOTAGA	Sulfo-Cy5	Sub-KuE structure	PSMA	[52]
**Shi, Xu et al.**	2022	Preclinical	Glioblastoma	PET/NIR-II	^64^Cu	DOTA	ICG	Folic acid	Folate receptor	[78]
**Sun, Ding et al.**	2016	Preclinical	Glioblastoma	PET/Fluorescence	^68^Ga	NOTA	Pyrazoline	AE105	UPAR	[38]
**Sun, Zeng et al.**	2018	Preclinical	Glioblastoma	PET/NIR-II	^68^Ga	NOTA	CH1055	c(RGDfK)	Integrin α_v_β_3_	[71]
**Wang, Mao et al.**	2017	Preclinical	Ovarian cancer	PET/NIR-I	^64^Cu	DOTA	IRDye800	15D3	P-glycoprotein	[20]
**Yang, Zhao et al.**	2021	Preclinical	Brain and breast cancers	PET/NIR-I	^89^Zr	DFO	IRDye78	Passive targeting	Passive targeting	[105]
**Yu, Huang et al.**	2023	Preclinical	Breast cancer	PET/NIR-II	^68^Ga	DOTA	TPA-TTINC	Passive targeting	Passive targeting	[36]
**Yu, Wei et al.**	2018	Preclinical	Breast cancer	PET/fluorescence	^177^Lu	N/A	mTCPP	Passive targeting	Passive targeting	[107]
**Zettlitz, Tsai et al.**	2018	Preclinical	Pancreatic cancer	PET/NIR-I	^124^I	N/A	IRDye800CW	A2cDb	Prostate stem cell antigen	[92]
**Zettlitz, Waldmann et al.**	2019	Preclinical	Prostate cancer	PET/fluorescence	^18^F	Tetrazine	Sulfo-Cy5.5	A2cDb	Prostate stem cell antigen	[93]
**Zhang, Desai et al.**	2017	Preclinical	Prostate cancer	PET/fluorescence	^68^Ga	DOTA	IRDye650	HZ219	GRP receptor	[73]
**Zhang, Hong et al.**	2012	Preclinical	Breast cancer	PET/NIR-I	^64^Cu	NOTA	IRDye800CW	TRC105	CD105	[62]
**Zhang, Hong et al.**	2013	Preclinical	Breast cancer experimental lung metastasis	PET/NIR-I	^64^Cu	NOTA	IRDye800CW	TRC105	CD105	[61]
**Zhang, Huang et al.**	2024	Preclinical	Lung cancer	PET/fluorescence	^68^Ga	DOTA	FAM	FAPI-04	FAP	[80]
**Zhou, He et al.**	2023	Preclinical	Breast cancer, cervical cancer, glioblastoma and hepatocellular cancer	PET/NIR-I	^68^Ga, ^90^Y	DOTA	Sulfo-Cy5.5	Cbz-Phe-Lys-AOMKs	Cysteine cathepsin B	[85]

### Emerging techniques and strategies for optimizing PET/fluorescence probes

With the ongoing development of new materials and technologies, the continuous optimization of dual-modality probes further advances the precision of tumor diagnosis and treatment. QDs are a distinct class of engineered nanomaterials with exceptional optoelectronic properties, positioning them as a highly promising alternative to traditional luminescent dyes in various biomedical applications, such as biomolecule targeting, luminescence imaging, and drug delivery [96]. Cai et al. [97] quantitatively evaluated the tumor targeting efficacy of QD-based bifunctional probes. Chen [98] et al. used amine-functionalized QD for VEGFR-targeted PET/NIRF imaging. Both studies demonstrate the advantages of this method in targeted imaging of deep tissues. Luo et al. [99] designed a dual-target probe targeting CD105 and TF and verified its higher targeting efficiency in a pancreatic cancer model. Hu et al. [100] combined QD with dual-target PET/fluorescence probes, opening up a new strategy for the development of multitarget multimodal probes with higher tumor-targeting efficiency.

Some dual-modality probes do not have an active targeting group. They either utilize the tumor affinity of fluorophores or utilize nanomaterials for passive targeting [101, 102]. In addition, multifunctional nanoparticles have both diagnostic and therapeutic functions, which can be guided by PET/FLI for photodynamic or photothermal cancer therapy [36, 43]. Although passive targeting mediated by nanomaterials may have the problem of nonspecific accumulation, its wide applicability will greatly promote the integration of diagnosis and treatment of a variety of cancers.

The introduction of specific molecules can alter certain properties of dual-modality probes. Lwin et al. [44] utilized PEG to link the dye to the antibody, improving the water solubility and biocompatibility of the probe and reducing nonspecific binding. Rodriguez et al. [103] achieved solid phase synthesis and release of the probe by biotin-modified dioxyboron-heterocycle, which improved the specific activity of the probe. Hu et al. [104] designed self-emitting gold nanoclusters that produced satisfactory self-emitting images of tumors in the absence of external excitation. Yang et al. [105] modulated the clearance pathway with a generalized strategy of beneficial renal clearance by introducing α-LA.

Ariztia et al. improved the flexibility and diversity of probe synthesis by introducing different groups through click chemistry based on a C-glycosidyl skeleton capable of integrating multiple functional groups. At the same time, PEG chain was introduced into the probe to improve the stability of the probe *in vivo* and reduce nonspecific binding [106]. Yu et al. developed size-controlled porphyrin-PEG nanocomplexes for dual tumor-mitochondrial targeted therapy, which can be combined with PDT to achieve accurate combined tumor diagnosis and treatment based on dual-modality probes [107]. Using chimeric antigen receptor (CAR) T cells as the core, Harmsen et al. constructed a PET/fluorescence dual-modality probe that can perform long-term whole-body CAR T-cell tracking in an ovarian peritoneal cancer model. It reflects the advantages of the precision diagnosis and treatment of cancer [108]. In the diagnosis and treatment of brain tumors, Pang et al. developed a dual-imaging monitored virus-like nanotherapeutic agent. Convection-enhanced delivery is used to achieve brain tumor treatment and image tracking with good results [109].

Recent advancements in material science have significantly propelled the development of dual-modality imaging probes by introducing a wide array of novel nanomaterials with enhanced physicochemical and biological properties. Engineered platforms such as QDs, mesoporous silica-coated upconversion nanoparticles, and dendrimer-based hybrid probes have demonstrated improved biocompatibility, tumor-targeting specificity, and multifunctionality [110–112]. These materials enable precise integration of diagnostic and therapeutic functions, responsiveness to TMEs, and controlled clearance profiles. In addition, molecular engineering strategies—including click chemistry, PEGylation, and surface functionalization—further optimize probe stability, targeting efficiency, and *in vivo* performance [113]. The development of stimuli-responsive, biodegradable, and clinically translatable nanocarriers, as well as integration with emerging technologies like CAR-T tracking and virus-like delivery systems, will drive the next generation of intelligent dual-modality probes, accelerating the realization of precision cancer diagnosis and therapy.

### Advantages of PET/FLI probes

At present, ^18^F-FDG PET/CT is commonly used in clinical tumor diagnosis [114]. The emergence of PET/fluorescence dual-modality probes has successfully improved tumor targeting and imaging sensitivity and resolution. The combination of the two imaging methods realizes the integrated diagnosis and treatment of preoperative diagnosis and intraoperative fluorescence navigation, which has better convenience and consistency [91]. In addition, the flexible structure of the dual-mode probe also allows the introduction of a variety of groups to adjust the performance of the probe, which can better serve the clinical application [105]. Such dual-modality probes can also combine imaging with PDT or photothermal therapy (PTT), further realizing the combination of diagnosis and treatment [36, 58].

As numerous studies have reported, intraoperative NIR-I FLI enables accurate real-time tumor delineation. However, the penetration depth of emitted light in biological tissue is limited. Developing novel NIR-II dual-modality molecular probes for *in vivo* multimodal imaging applications is therefore of significant importance and has a direct impact on the field of biomedicine.

**Figure 4.**
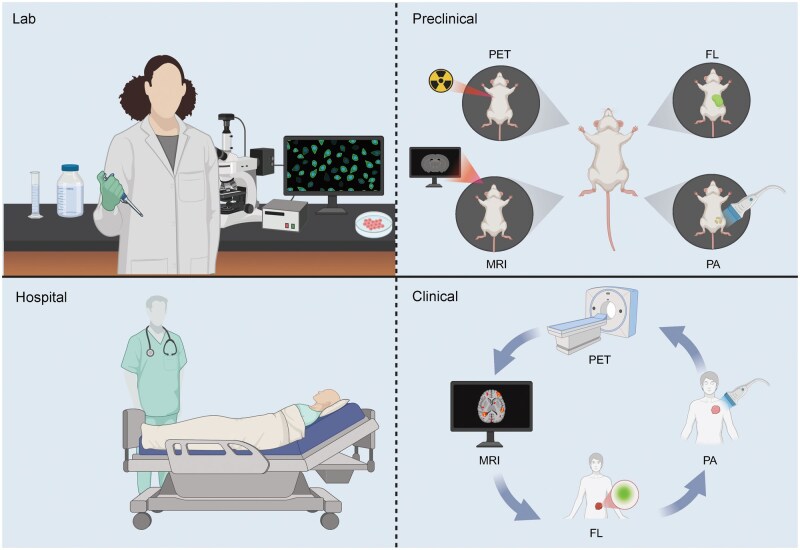
**The translation of dual-modality probes from preclinical research to clinical application.** The effectiveness and safety of probe imaging are validated through preoperative imaging and intraoperative fluorescence guidance in mice, thereby advancing the translation to clinical application. FL, fluorescence imaging; PA, photoacoustic imaging. This figure was created with BioRender.

In summary, the PET/fluorescence dual-modality molecular probes showed excellent performance [115]. Its diversity and strong targeting capabilities also enable personalized, precise tumor diagnosis and treatment. However, the translational pathway of dual-modality probes from preclinical research to clinical application still requires significant enhancement (Fig. 4). With a deeper understanding of tumor molecular mechanisms and advancements in related technologies, PET/fluorescence dual-modality probes hold promising development prospects.

## MRI/fluorescence dual-modality molecular imaging

### Fundamentals of MRI/fluorescence dual-modality imaging

Magnetic resonance imaging/fluorescence dual-modality imaging integrates the high spatial resolution and deep tissue penetration of MRI with the high sensitivity and molecular specificity of FLI. MRI imaging is based on the interaction of hydrogen nuclei with an external magnetic field and radiofrequency (RF) pulses. When placed in a strong magnetic field, hydrogen protons align along the field direction. RF pulses temporarily disturb this alignment, and as the protons relax back to equilibrium, they emit signals that are detected and processed to generate high-resolution images. The contrast in MRI depends on *T*₁ (longitudinal) and *T*₂ (transverse) relaxation times, which vary among tissues [116].

Magnetic resonance imaging contrast is achieved using exogenous agents such as paramagnetic (Gd³^+^ complexes) or superparamagnetic (SPIONs) materials, which modulate proton relaxation times to enhance image contrast [117]. FLI enables real-time, high-sensitivity visualization of molecular interactions at the cellular level. The combination of these modalities allows for precise tumor localization and characterization, offering both anatomical and functional insights essential for precision oncology.

### Applications of MRI/fluorescence dual-modality probes in tumor diagnosis

Accurate and early tumor diagnosis is essential for effective treatment and improved patient outcomes. Traditional single-modality imaging techniques, such as MRI or FLI, often face limitations in sensitivity, spatial resolution, or depth penetration. To overcome these challenges, MRI/fluorescence dual-modality probes have emerged as powerful tools that combine the high spatial resolution of MRI with the superior sensitivity and molecular specificity of FLI [118]. These probes offer complementary advantages, enabling precise tumor localization, real-time tracking, and enhanced contrast in deep-seated lesions (Table 2).

**Table 2.** Summary of key information on MRI/fluorescence dual-modality probes in oncology.

Author	Year	Type of cancer	Imaging technologies	Magnetic material	Dye	Targeting group	Receptor		Reference
**Chen, Liu et al.**	2019	Hepatocellular carcinoma	MRI/NIR-I	Gd	Porphyrin	Folic acid	Folate receptor		[157]
**Cheng, Kong et al.**	2024	Osteosarcoma	MRI/NIR-II	mCu&Ce	ICG	RGD	Integrin α_v_β_3_		[165]
**Dong, Ye et al.**	2022	Breast cancer	MRI/NIR-I	Au/Gd@BSA NCs	Au/Gd@BSA NCs	Folic acid	Folate receptor		[150]
**Du, Liang et al.**	2018	Breast cancer and colon cancer	MRI/NIR-I	Gd	IRDye800CW	PD-L1 antibodies	PD-L1		[170]
**Duan, Zhang et al.**	2023	Breast cancer	MRI/NIR-II	Gd	Gd: Nd-RENPs	Passive targeting	Passive targeting		[147]
**Fang, Yang et al.**	2022	Breast cancer	MRI/NIR-I	Fe	Porphyrin	Folic acid	Folate receptor		[163]
**Fu, Fu et al.**	2022	Breast cancer	MRI/NIR-I	Mn	ICG	Passive targeting	Passive targeting		[132]
**Guan, Liang et al.**	2017	Oral squamous cell carcinoma	MRI/Fluorescence	Gd	FITC	Folic acid	Folate receptor		[169]
**Guan, Zeng et al.**	2024	Breast cancer	MRI/NIR-II	Mn	IR780	Passive targeting	Passive targeting		[161]
**Guo, Song et al.**	2020	Colon cancer	MRI/NIR-I	Fe₃O₄	Cy5.5	Poly-l-lysine	Trypsin		[135]
**Han, Zhang et al.**	2020	Breast cancer	MRI/fluorescence	Fe₃O₄	CQDs	CD44 mAb	CD44		[133]
**Huang, Chen et al.**	2017	Cervical cancer	MRI/fluorescence	Gd	NaGdF4: Eu	Folic acid	Folate receptor		[119]
**Huang, Fan et al.**	2018	Pancreatic cancer	MRI/fluorescence	Gd	Gd-Au NCs	Glypican-1 antibody	Glypican-1		[120]
**Li, Cao et al.**	2015	Glioblastoma	MRI/NIR-II	Gd	Ag_2_S QDs	Passive targeting	Passive targeting		[146]
**Li, Lee et al.**	2020	Lung cancer	MRI/Fluorescence	Gd	TPE-BPA	Transferrin	Transferrin receptor		[174]
**Li, Li et al.**	2024	Lung cancer	MRI/NIR-II	SPIONs	IRDye800CW	Cetuximab	EGFR		[144]
**Li, Li et al.**	2024	Melanoma	MRI/NIR-I	Fe₃O₄	IR820	Chitosan	N/A		[162]
**Li, Peng et al.**	2022	N/A	MRI/NIR-I	^19^F	Aza-BODIPYs	Passive targeting	Passive targeting		[158]
**Li, Wang et al.**	2018	N/A	MRI/fluorescence	SPIONs	Nile red	mPEG-Lys3-CA4	N/A		[131]
**Li, Xue et al.**	2022	Hepatocellular carcinoma	MRI/NIR-I	Gd	DCDSTCY	Passive targeting	Passive targeting		[139]
**Liu, Zhang et al.**	2023	Breast cancer	MRI/NIR-II	Gd	ICG	Atezolizumab	PD-L1		[173]
**Ma, Yan et al.**	2020	Lung cancer	MRI/NIR-I	IONPs	NQ-Cy	Folic acid	Folate receptor		[151]
**Mei, Wang et al.**	2018	Hepatocellular carcinoma	MRI/fluorescence	Gd	AuNCs	Passive targeting	Passive targeting		[154]
**Olson, Jiang et al.**	2010	Breast cancer and fibrosarcoma	MRI/NIR-I	Gd	Cy5	ACPPs	MMP-2, MMP-9		[142]
**Qin, Peng et al.**	2019	Breast cancer	MRI/NIR-I	Si−Gd nanoparticles	Ce6	Folic acid	Folate receptor		[171]
**Ravoori, Singh et al.**	2016	Ovarian cancer	MRI/NIR-I	Gd	ICG	Passive targeting	Passive targeting		[140]
**Ren, He et al.**	2020	Hepatocellular carcinoma	MRI/NIR-II	Gd	Gd-REs	Passive targeting	Passive targeting		[145]
**Ren, Song et al.**	2021	Pancreatic cancer	MRI/NIR-I	Fe₃O₄	Cy7	Passive targeting	Passive targeting		[167]
**Sang, Gao et al.**	2022	Colon cancer	MRI/NIR-I	SPIONs	IR780	Peptide ACKFRGD	Integrin receptors		[164]
**Shen, Li et al.**	2017	Cervical cancer and breast cancer	MRI/fluorescence	Fe₃O₄	CdSe/ZnS QDs	Folic acid	Folate receptor		[168]
**Song, Shen et al.**	2017	Breast cancer	MRI/NIR-I	IONPs	ICG	Passive targeting	Passive targeting		[153]
**Su, Chan et al.**	2017	Cervical cancer	MRI/fluorescence	Fe₃O₄	GQD	Folic acid	Folate receptor		[45]
**Tang, Zhu et al.**	2022	Lung cancer	MRI/NIR-I	Fe	Aza-BODIPYs	Passive targeting	Passive targeting		[134]
**Wang, Huang et al.**	2021	Hepatocellular carcinoma	MRI/NIR-I	N/A	Cy5.5	cRGD	Integrin α_v_β_4_		[136]
**Wang, Huo et al.**	2022	Breast cancer	MRI/fluorescence	Fe₃O₄	Cy5	P0 aptamer	MUC1 protein		[138]
**Wang, Li et al.**	2021	Breast cancer	MRI/NIR-I	Gd_2_O_3_	AuNCs	AS1411 aptamers	Nucleolin		[155]
**Wang, Mao et al.**	2020	Breast cancer	MRI/NIR-I	SPIONs	IR780	Passive targeting	Passive targeting		[152]
**Wang, Wan et al.**	2021	Breast cancer	MRI/fluorescence	Gd	N-DO3AtBu	Passive targeting	Passive targeting		[121]
**Wu, Liu et al.**	2021	Head and neck squamous cell carcinomas	MRI/NIR-II	Gd	NaGdF_4_	cMBP	cMet receptor		[122]
**Xiao, Cai et al.**	2023	Breast cancer	MRI/fluorescence	Gd	TPBP	Passive targeting	Passive targeting		[123]
**Xie, Hao et al.**	2024	Thyroid cancer	MRI/NIR-I	MnO_2_	Cy5.5	BSA	Albumin receptor gp60 and secreted protein acidic and rich in cysteine	[137]	
**Xu, Jia et al.**	2014	Glioblastoma	MRI/fluorescence	Gd	CQDs	RGD	Integrin α_v_β_3_		[127]
**Xu, Zang et al.**	2023	Cervical cancer	MRI/fluorescence	Gd	Cu-In-S	Passive targeting	Passive targeting		[128]
**Yang, Huang et al.**	2024	Gastric cancer	MRI/NIR-I	Gd	Cy7	CREKA peptide	Fibronectin		[124]
**Yu, Ding et al.**	2023	Breast cancer	MRI/NIR-I&II	Gd	ICG	Albumin	Albumin receptor gp60	[156]	
**Zhang, Deng et al.**	2022	Hepatocellular carcinoma	MRI/fluorescence	SPIONs	TPE	SP94 peptide	GRP78 protein		[172]
**Zhang, Li et al.**	2017	Breast cancer	MRI/NIR-I	Fe₃O₄	Porphyrin	Passive targeting	Passive targeting		[160]
**Zhang, Shu et al.**	2022	Breast cancer	MRI/NIR-II	SPIONs	DCNP	CREKA peptide	Fibronectin		[166]
**Zhang, Wu et al.**	2022	Hepatocellular carcinoma	MRI/fluorescence	Fe₃O₄	CQDs	Passive targeting	Passive targeting		[130]
**Zhang, Zhang et al.**	2014	Breast cancer	MRI/NIR-I	IONPs	Cy5.5	Anti-EGFR mAb	EGFR		[129]
**Zhao, Fan et al.**	2014	Leukemia	MRI/fluorescence	MnO_2_	Cy5	Aptamer	PTK7		[31]
**Zhao, Zhao et al.**	2020	Glioblastoma	MRI/fluorescence	Gd	RFP	RGD	Integrin α_v_β_3_		[126]
**Zheng, Jia et al.**	2021	Breast cancer	MRI/NIR-II	MnO_2_	CQ4T	Passive targeting	Passive targeting		[159]
**Zheng, Tang et al.**	2022	Melanoma	MRI/fluorescence	Gd	RhB	YIGSR and RGD	Integrin α_v_β_3_ and laminin receptor	[125]	
**Zheng, Zhang et al.**	2025	Glioblastoma	MRI/NIR-I	Gd	ICG	DC-G16-11	GCN5	[141]	
**Zou, Chen et al.**	2024	Breast cancer	MRI/NIR-II	Gd	Ag_2_S QDs	Cetuximab	EGFR		[143]

Gadolinium (Gd)-based magnetic materials are often used in MRI/fluorescence dual-modality probes to improve the signal intensity of *T*₁ weighted imaging. Huang et al. [119] developed an MRI/fluorescent dual-modality probe (FA-PEI-NaGdF_4_: Eu nanoparticles) targeting cancer cells with a large number of folate receptors with high biocompatibility, excellent imaging performance, and excellent targeting. Huang et al. [120] used Glypican-1 antibody conjugated with Gd–Au nanoclusters in the probe for targeted diagnosis of pancreatic cancer. Wang et al. present a facile strategy using albumin aggregates to achieve signal enhancement [121]. Wu et al. [122] provided novel and effective diagnostic methods for head and neck squamous cell carcinoma by means of dual-modality *T*_1_ weighted MRI and NIR-II luminescence imaging methods. A magnetic resonance (MR)/two-photon fluorescence dual-modal contrast agent, Gd-DOTA-TPBP, was constructed by Xiao et al. using an amphiphilic block copolymer from oligo(ethylene glycol) methyl ether methacrylate and N-(2-hydroxypropyl) methacrylamide derivatives as the carrier. Compared with MRI, it has higher resolution and higher sensitivity [123]. Fibronectin-targeting magnetic resonance/near-infrared fluorescence (MR/NIRF) imaging contrast agents enabled effective noninvasive detection of gastric cancer and its metastases, demonstrating potential for improved clinical diagnosis [124]. Zheng et al. [125] and Zhao [126] et al. also constructed MRI/fluorescence dual-modality probes with good imaging effect, which have potential application in a variety of cancers. It is worth mentioning that QDs are often used as fluorophores in probes, and Gd^3+^ interference can be effectively avoided by using ZnS shell [127, 128].

In addition to Gd, elements such as Mn and Fe are also often used as the basis of magnetic materials in MRI/fluorescence probes [31, 129, 130]. Li et al. [131] used novel SPIONs and Nile red co-loaded mPEG-Lys3-CA4-NR/SPION polymer micelles to label Raw264.7 cells, which can be used as a new lymphatic contrast agent. Similarly, Fu et al. prepared F127-ICG/Mn nanoparticles as fluorescent/MR dual-modality probes can be effectively used for the diagnosis of sentinel lymph node (SLN) metastasis [132]. The MCNPs-CD44 probe developed by Han et al. [133] can detect as low as a few hundred cancer cells for breast cancer with extremely high resolution. Tang et al. [134] prepared a ^19^F MRI–FLI dual-modality nanoemulsion for dual-imaging tracking of lung cancer A549 cells and macrophages. Guo et al.’s [135] probe can dissociate in response to the hydrolysis of trypsin, thereby significantly enhancing the NIRF signal (approximately 18-fold) and altering the MR signal for dual-modality imaging of pancreatic cancer. Wang et al. [136] developed dual-mode MRI and NIRF probes to detect early-stage hepatocellular carcinoma by targeting integrin α_v_β_3_. Xie et al. [137] prepared a novel pH-responsive MR/NIRF nanoprobe for accurate diagnosis of thyroid cancer. The dual-modality probe prepared by Wang et al. [138] can selectively capture/enrich circulating tumor cells (CTCS) due to its stable targeting, unique magnetic properties, and regulatory interactions between quenched groups and fluorophores, thus achieving sensitive CTC detection/imaging even in blood.

By integrating the strengths of MRI and FLI, researchers have developed a range of dual-modality probes for the diagnostic imaging of primary and metastatic tumors. The distinctive dual-modality structure enhances specificity, improves resolution, and delivers superior imaging performance compared to single-modality techniques. However, current research remains confined to preclinical studies, and the clinical efficacy of MRI/fluorescence dual-modality probes still requires validation through clinical trials.

### MRI/fluorescence dual-modality probes for intraoperative navigation

Intraoperative fluorescence navigation has emerged as a key application of FLI in recent years. When combined with MRI, it offers real-time anatomical information, along with enhanced tissue penetration and resolution, surpassing the capabilities of single-modality navigation. A number of studies have shown that intraoperative navigation guided by MRI/fluorescence dual-modality probes can achieve accurate resection of hepatocellular carcinoma, ovarian cancer and other cancers, which has great clinical application potential [139, 140]. Recently, Zheng et al. developed a dual-modality probe targeting glioblastoma (GBM) via GCN5 molecular recognition. This probe enables both MRI and FLI, allowing for precise intraoperative tumor resection under fluorescence guidance by integrating preoperative boundary data. The approach demonstrates strong potential to reduce postoperative recurrence rates [141]. Probes developed by Olson et al. are capable of detecting residual tumors and metastases as small as 200 microns, which can then be excised under fluorescence guidance. Additionally, activated Gd-labeled nanoparticles are efficiently deposited within the tumor parenchyma, enhancing MRI-guided staging and preoperative planning [142].

NIR-II imaging outperforms NIR-I imaging in terms of resolution, background interference, and tissue penetration, which has garnered significant attention from scientists in the field of MRI/FLI [143, 144].

Localization of small lesions and determination of diffuse boundaries are important challenges in the diagnosis and treatment of hepatocellular carcinoma. Ren et al. [145] reported an MRI/NIR-II probe Gd-REs@Lips, which can visualize small lesions (2 mm) on the liver surface and is expected to fill the gap between preoperative detection and intraoperative guidance. MRI is commonly used for the diagnosis of brain tumors. In view of this, Li et al. [146] clearly delineated the brain tumor with the Gd-Ag_2_S nanoprobe and accurately completed the intraoperative resection of the tumor in the mouse model, which has broad application prospects. Duan et al. [147] prepared rare-earth nanoparticles (Gd: Nd-RENV) with NIR-II fluorescence and MRI properties that can rapidly distinguish metastatic SLNs from normal lymph nodes and guide precise surgical resection of metastatic lymph nodes.

Magnetic resonance imaging/fluorescence dual-modality probe not only improves the effect of intraoperative navigation in terms of resolution and penetration ability but also makes the two imaging methods have good consistency and stability [147]. By combining diagnosis with intraoperative guided resection, MRI/Fluorescence probes can better realize tumor integration and have broad application prospects.

### MRI/fluorescence dual-modality probes in photodynamic, photothermal, and chemodynamic therapy

Photodynamic therapy, PTT, and chemodynamic therapy (CDT) are innovative therapeutic strategies that leverage external stimuli, such as light or chemical reactions, to selectively target and eliminate tumor cells. Each of these therapies relies on distinct mechanisms: PDT utilizes photosensitizers activated by light to generate reactive oxygen species (ROS), PTT employs light-absorbing nanoparticles to induce localized heat and destroy tumor cells, and CDT generates cytotoxic hydroxyl radicals in response to the tumor’s unique microenvironment [148–151]. The integration of MRI and fluorescence dual-modality probes into these therapies has shown great potential in improving the precision and efficacy of such treatments [152–154].

Many MRI/fluorescence dual-modality probes combine imaging with PDT, PTT, or CDT. Wang et al. coupled the prepared Au nanobipyramidal with Gd_2_O_3_, Au nanoclusters, and denatured bovine serum albumin (aunBP-Gd_2_O_3_/Au-DBSA) and developed a platform that could be used for MRI/fluorescence dual-modality image-guided PTT. It showed excellent photothermal anticancer effect of over 95% *in vitro* and *in vivo* [155]. Yu et al. [156] developed a probe GD-EB-ICG (GI) that self-assembled with endogenous albumin and significantly improved fluorescence quantum yield and photothermal conversion efficiency. Chen et al. [157] used a dual-modality probe targeting the folate receptor to achieve excellent imaging and clearance of hepatocellular carcinoma tumor cells by PDT. Li et al. [158] found that the selective introduction of trifluoromethyl (CF3) groups into aza-BODIPYs significantly improved UV absorption, fluorescence emission, photothermal efficiency, and ROS generation performance. Zheng et al. used a novel TME-activated nanosystem (FMSN-MnO_2_-BCQ) for MRI/NIR-II imaging and self-platelet elimination of tumor cells. Under this positive feedback mechanism, the probe achieved a high killing effect [159].

It is worth mentioning that MRI/fluorescence probes can integrate multiple therapeutic approaches [160, 161]. Li et al. developed a multifunctional nanosystem MNPs/GOD@CS/IR820, which synergistically integrates CDT and PDT. It can release active ingredients in response to weakly acidic TME, alleviate the limitation of hypoxia and endogenous H₂O₂ deficiency on PDT and CDT, and has a significant antitumor effect [162]. Fang et al. developed nanoplatforms based on graphene oxide (GO) and metal-organic framework (MOF) Fe-porphyrin, in which the MOF can act as a photosensitizer to trigger PTT and PDT. This nanoplatform allows precise targeting of cancer cells, avoiding immune elimination and prolonging blood circulation [163]. The integration of PTT with immunotherapy in the probe by Sang et al. [164] provides a promising nanotherapeutic strategy for future cancer treatment. The novel dual-modality probe developed by Cheng et al. [165] can be used for precise targeting and effective ablation of osteosarcoma (OS) by PTT-enhanced CDT and subsequent *in vitro* and *in vivo* immunogenetic cell death stimulation.

By combining with a variety of therapeutic modalities, the application range of MRI/fluorescence dual-modality probes is further broadened. The combined capabilities of these imaging techniques can more accurately target tumors, enhance treatment, and enable monitoring and assessment, thereby improving the overall therapeutic efficacy of PDT, PTT, and CDT.

### Other applications of MRI/fluorescence dual-modality probes

Magnetic resonance imaging/fluorescence dual-modality probes also play an important role in other aspects of tumor diagnosis and treatment. Some dual-modality probes can be used to deliver chemical drugs [45, 166, 167]. For instance, Shen et al. [168] employed probes to deliver doxorubicin (DOX) along with vascular endothelial growth factor (VEGF)-targeted small hairpin RNA (shRNA) into tumor cells, demonstrating a pronounced synergistic antitumor effect. Similarly, Guan et al. [169] designed a dual-modality probe targeting the folate receptor and used it as a carrier of DOX for chemotherapy. Du et al. [170] produced nanohybrid liposomal neural membrane nanoparticles loaded with the chemotherapeutic drug paclitaxel to achieve precise tumor killing by targeting PD-L1. The porous structure of Qin et al.’s [171] dual-modality probe loaded with DOX was used for chemotherapy, and chlorine e6 (Ce6) was excited by NIR radiation for PDT, which effectively reduced tumor volume.

Magnetic resonance imaging/fluorescence probes can also be combined with emerging technologies or applications, which has broad research prospects. Zhang et al. [172] loaded survivin small interfering RNA into nanoparticles to construct a dual-modality probe capable of gene therapy to inhibit tumor growth. Liu et al. [173] developed nanoparticle atezolizumab (NPs-Ate) to target PD-L1 for immunotherapy guided by MRI/NIR-II imaging and monitored in real time. Li et al. [174] used programmed self-assembly technology to construct nanoparticles with dual-modality imaging and therapeutic functions, which can effectively kill cancer cells through highly positive charges and heavy toxicity of nanoparticles.

Whether for diagnosis, surgical resection, or various treatment modalities, MRI/fluorescence dual-modality probes demonstrate superior performance and seamlessly integrate these applications. Their exceptional resolution, enhanced tissue penetration, and ability to combine diagnosis and treatment make MRI/fluorescence dual-modality probes indispensable for the precise diagnosis and treatment of tumors, offering significant advantages over single-modality probes. However, it is important to acknowledge that MRI/fluorescence dual-modality probes have certain limitations, including instability in imaging, high background signals and significant difference in sensitivity. Additionally, these probes remain in the preclinical research phase, and the inherent limitations of MRI may hinder their effectiveness in certain cancers, such as lung cancer. Consequently, further investment in exploring diverse application scenarios and combined modalities is essential.

## Photoacoustic/fluorescence dual-modality molecular imaging

### Mechanism of photoacoustic/fluorescence dual-modality imaging

Photoacoustic imaging (PA) represents an innovative biomedical imaging modality that integrates the advantages of optical and ultrasonic imaging. In this technique, tissues are irradiated with short-pulse laser beams, leading to the absorption of light energy and subsequent thermoelastic expansion. This process generates ultrasonic waves, which are captured by an array of transducers [175, 176]. By combining the high spatial resolution of ultrasound with the rich functional information derived from optical absorption, PA imaging offers a unique capability to achieve high-resolution imaging at significant tissue depths (up to several centimeters) [177]. This depth penetration is facilitated by the superior propagation characteristics of sound waves in biological tissues compared to light.

The integration of photoacoustic and fluorescence imaging combines the advantages of both modalities. Photoacoustic imaging allows for deep tissue penetration and spatial resolution, while FLI offers high sensitivity and molecular specificity at shallower depths [178]. By combining both, a dual-modality probe can provide complementary information for precise diagnosis and treatment of a variety of cancers [179–184] (Table 3). This synergy enhances the accuracy of tumor detection, diagnosis, and monitoring of treatment response, and it has broad application prospects in intraoperative navigation and various treatments.

**Table 3.** Summary of key information on PA/fluorescence dual-modality probes in oncology.

Author	Year	Type of cancer	Imaging technologies	Photoacoustic contrast agents	Dye	Targeting group	Receptor	Reference
**Bi, Deng et al.**	2021	Colorectal cancer	PA/NIR-II	Ag_2_S	Ag_2_S	Passive targeting	Passive targeting	[226]
**Chao, Zhang et al.**	2024	Cervical cancer and hepatocellular carcinoma	PA/NIR-I	Xanthene	Xanthene	Passive targeting	Passive targeting	[181]
**Chen, Gao et al.**	2018	Esophageal adenocarcinoma	PA/NIR-I	IRDye800CW	IRDye800CW	QRH* and KSP*	EGFR and HER2	[227]
**Chen, Liu et al.**	2016	Cervical cancer	PA/NIR-I	ICG	ICG	Passive targeting	Passive targeting	[207]
**Chen, Wang et al.**	2022	Hepatocellular carcinoma	PA/NIR-I	DCI	DCI	Acryloyl group	Cys	[215]
**Chen, Zhao et al.**	2016	Breast cancer	PA/NIR-I	ICG	ICG	EpCAM, N-cadherin and galectin-3	EpCAM, N-cadherin and galectin-3	[193]
**Chen, Zuo et al.**	2021	Breast cancer	PA/NIR-I	ICG	ICG	Passive targeting	Passive targeting	[198]
**Cheng, Chen et al.**	2017	Thyroid cancer	PA/NIR-II	CH1000	CH1000	Ac-Cys-ZEGFR:1907	EGFR	[229]
**Dai, Yu et al.**	2020	Breast cancer	PA/NIR-I	DiR-BOA	DiR-BOA	HA and HPPS	CD44 and SR-B1	[203]
**Doan, Nguyen et al.**	2021	Breast cancer	PA/NIR-I	IR783	IR783	Passive targeting	Passive targeting	[194]
**Fan, Jiang et al.**	2024	Breast cancer	PA/NIR-I	IRDye800CW	IRDye800CW	Mannose	CD206	[205]
**Gao, Ma et al.**	2019	Cervical cancer	PA/NIR-I	LET-CyOH	LET-CyOH	Phosphate group	Alkaline phosphatase	[208]
**Ge, Fu et al.**	2020	Breast cancer	PA/NIR-II	AuNR	IR1061	Passive targeting	Passive targeting	[192]
**Gu, Yang et al.**	2023	Cervical cancer and hepatocellular carcinoma	PA/NIR-I	Cyanine dye	Cyanine dye	Biotin	Biotin receptor	[182]
**Guan, Hu et al.**	2023	Breast cancer	PA/NIR-I	Rhodamine	Rhodamine	Passive targeting	Passive targeting	[179]
**Guan, Shang et al.**	2017	Hepatocellular carcinoma	PA/NIR-I	AuNR	ICG	Passive targeting	Passive targeting	[216]
**Guo, Feng et al.**	2019	Glioblastoma	PA/NIR-II	PBT	PBT	Passive targeting	Passive targeting	[220]
**Guo, Li et al.**	2021	Cervical cancer	PA/NIR-II	B4C@C	B4C@C	Passive targeting	Passive targeting	[210]
**Hu, Liu et al.**	2024	Pancreatic cancer	PA/NIR-I	SHD	SiRho and SHD	4-Nitrobenzyl bromide	NTR	[223]
**Kwon, Kim et al.**	2022	Breast cancer	PA/NIR-I	Hexa-BODIPY	Hexa-BODIPY	Passive targeting	Passive targeting	[196]
**Li, Bottini et al.**	2019	Breast cancer	PA/NIR-I	CuS nanoparticles	ICG	Passive targeting	Passive targeting	[40]
**Li, Liu et al.**	2017	Breast cancer	PA/NIR-I	ICG	ICG	Passive targeting	Passive targeting	[199]
**Li, Peng et al.**	2018	Breast cancer	PA/NIR-I	IR780	IR780	Passive targeting	Passive targeting	[200]
**Li, Wang et al.**	2021	Breast cancer and hepatocellular carcinoma	PA/NIR-II	BBTD-BET	BBTD-BET	Passive targeting	Passive targeting	[184]
**Li, Xiu et al.**	2021	Breast cancer	PA/NIR-I	MoS2 nanosheets	Cy7	Passive targeting	Passive targeting	[187]
**Li, Zhang et al.**	2024	Pancreatic cancer	PA/NIR-I	S-HDs	S-HDs	Acrylate functional group and 2,4-dinitrobenzenesulfonyl group	Cys and GSH	[224]
**Lin, Liu et al.**	2018	Breast cancer	PA/NIR-I	ICG	ICG	Passive targeting	Passive targeting	[191]
**Long, Yang et al.**	2023	Lung cancer	PA/NIR-II	DTTB	DTTB	Passive targeting	Passive targeting	[222]
**Lyu, Lu et al.**	2024	Glioblastoma	PA/NIR-II	IR-II-dye 5H5	IR-II-dye 5H5	cRGDfK	Integrin α_v_β_3_	[219]
**Park, Park et al.**	2020	Melanoma	PA/NIR-I	BSQ	BSQ	cRGD2	Integrin α_v_β_3_	[228]
**Peng, Shang et al.**	2018	Bladder cancer	PA/NIR-I	IRDye800CW	IRDye800CW	PLSWT7	CD44v6	[225]
**Saad, Grimaldo-Garcia et al.**	2024	Head and neck cancer	PA/NIR-II	SiNC(OH)	BPD	Cetuximab	EGFR	[214]
**Shakiba, Ng et al.**	2016	Head and neck cancer	PA/NIR-I	Bchl-lipid	Bchl-lipid	Passive targeting	Passive targeting	[211]
**Sheng, Guo et al.**	2018	Glioblastoma	PA/NIR-II	TB1	TB1	cRGD	Integrin α_v_β_3_	[218]
**Su, Yuan et al.**	2023	Breast cancer	PA/NIR-II	FEAA	FEAA	Passive targeting	Passive targeting	[197]
**Sun, Cai et al.**	2020	Laryngeal cancer	PA/NIR-II	Melanin nanoparticles(MNP)	H2	Passive targeting	Passive targeting	[212]
**Sun, Liang et al.**	2019	Breast cancer	PA/NIR-II	HCuS	fPEDC	cRGD	Integrin α_v_β_3_	[201]
**Sun, Zhao et al.**	2021	Colorectal cancer and ovarian cancer	PA/NIR-II	Ag_2_S QDs	Ag_2_S QDs	ZIGF-1	IGF-1R	[183]
**Wilson, Bachawal et al.**	2018	Breast cancer	PA/NIR-I	ICG	ICG	Anti-B7-H3 antibody	B7-H3	[202]
**Wu, Liu et al.**	2023	Breast cancer	PA/NIR-I	Hcy	Hcy	Biotin	Biotin receptor	[204]
**Xiang, Jiang et al.**	2023	Breast cancer	PA/NIR-I	Hcy	Hcy	Sulfate ester moiety	Sulfatase	[188]
**Xiao, Li et al.**	2017	Breast cancer	PA/NIR-I	MCDs	MCDs	Passive targeting	Passive targeting	[189]
**Xu, Jiao et al.**	2022	Neuroendocrine neoplasms	PA/NIR-II	C-NTBD nanoparticles and O-NTBD nanoparticles	C-NTBD nanoparticles and O-NTBD nanoparticles	Passive targeting	Passive targeting	[230]
**Xu, Liang et al.**	2024	Breast cancer	PA/NIR-II	Au nanoparticles	LDNPs	Passive targeting	Passive targeting	[190]
**Yang, Li et al.**	2018	Breast cancer and Hepatocellular carcinoma	PA/NIR-I	Cy5.5	Cy5.5	Passive targeting	Passive targeting	[180]
**Yuan, Diao et al.**	2022	Osteosarcoma	PA/NIR-II	PCPDTBT	PCPDTBT	PEGylated peptide PT	N/A	[232]
**Yuan, Fang et al.**	2022	Hepatocellular carcinoma	PA/NIR-I	AuNNPs	INT20	DEVD	Caspase-3	[217]
**Zhang, Kimura et al.**	2016	N/A	PA/NIR-I	Atto 740	Atto 740	R01 peptide	Integrin α_v_β_6_	[178]
**Zhang, Xu et al.**	2019	Breast cancer	PA/NIR-II	SYL	SYL	Passive targeting	Passive targeting	[185]
**Zhang, Zhang et al.**	2022	Lung cancer	PA/NIR-I	Methylene blue	NBD	Passive targeting	Passive targeting	[221]
**Zhang, Zhen et al.**	2018	Breast cancer	PA/NIR-I	CySO_3_OH	CySO_3_OH	Passive targeting	Passive targeting	[186]
**Zheng, Chen et al.**	2020	Laryngeal cancer	PA/NIR-II	Bi_2_S_3_-Ag_2_S	Bi_2_S_3_-Ag_2_S	Passive targeting	Passive targeting	[213]
**Zheng, Jia et al.**	2021	Breast cancer	PA/NIR-II	HSC-2	HSC-2	Passive targeting	Passive targeting	[195]
**Zou, Zhao et al.**	2023	Cervical cancer	PA/NIR-I	Cyanine dye	Cyanine dye	2,4-dinitrobenzenesulfonyl group	Cys	[209]

### Applications in diagnosis and treatment of various tumors

#### Breast cancer

PA/fluorescence dual-modality probes have been extensively studied and developed in breast cancer, which have shown excellent diagnostic effects [185, 186]. Li et al. [187] developed dual-modality probes for real-time imaging of endogenous furin activity with high sensitivity and selectivity. Xiang et al. [188] developed a dual-modality NIRF/PA probe for the first time to visualize sulfatase activity in animals and achieve accurate cancer diagnosis. Xiao et al. fabricated melanin carbonaceous dots (MCDs) that show great potential for fluorescence and photoacoustic dual-mode bioimaging. The probes can target triple-negative breast cancer tissue and therefore can be used for tumor dual-mode imaging [189]. Notably, a novel bubble-enhanced lanthanide-based dual-mode imaging nanoparticle with TME response was developed. Due to the bubble cavitation effect, the PA signal of LDAC nanoparticles can be enhanced with the generation of CO_2_ bubbles. As for the NIR-II fluorescence signal, it will also increase with the degradation of CaCO_3_. Such smart nanoparticles hold great promise for precision diagnostics in the future [190]. In addition to the primary tumor, lymph node metastasis of breast cancer and other metastatic lesions, such as lung metastasis, can also be detected and diagnosed by PA/fluorescence probes with high specificity and sensitivity [191].

Beyond diagnostic applications, PA/fluorescence dual-modality probes play a critical role in intraoperative navigation during breast cancer resection and in various therapeutic modalities [192]. Due to the inherent characteristics of PA/fluorescence probes, many probes themselves can also act as photothermal agents, facilitating the combination of imaging and PTT [193, 194]. Zheng et al. developed a NIR-II PA/NIR-II FLI-tunable nanoenzyme (HSC-2) to guide precise photothermal catalytic synergy therapy. The peroxidase mimetic activity of HSC-2 in the TME can be further enhanced by photothermal effects, and its catalase-like properties can eliminate excess ROS to protect normal cells, which has superior performance [195].

Similarly, the hexa-BODIPY-cyclotriphosphazene (HBCP) based nanoparticles developed by Kwon et al. [196] inhibited ROS generation while exhibiting excellent photothermal effects, thus allowing “safe” imaging. Su et al. [197] similarly achieved ablation of 4T1 tumors by PTT based on imaging with PA/fluorescence dual-modality probes. Moreover, by optimizing the probe structure, photothermal agents can be integrated with photodynamic agents into a single probe. The combined use of self-enhancing PTT and PDT to induce apoptosis in tumor cells under laser irradiation, guided by PA/FLI, opens new avenues for precision cancer therapy [198].

PA/fluorescence dual-modality probes can also achieve precise delivery of chemotherapy drugs. After the drug is delivered to the tumor site by the dual-modality probe, PA/fluorescence dual-modality imaging can monitor the drug release curve in real-time and noninvasively [40]. Scientists have also combined PA/fluorescence dual-modality imaging PTT with chemotherapy to efficiently induce the death of cancer cells. Li et al. [199] used the synergistic interaction of indocyanine green (ICG) and epirubicin to assemble small molecule nanoparticles and showed synergistic chemotherapeutic-PTT efficiency *in vivo*. Li et al. [200] constructed DTX/IR780 co-loaded mPEG-PCL micelles for PA/fluorescence dual-modality imaging-guided PTT/chemotherapy in breast cancer. In the future, PA/fluorescence-guided chemotherapy-phototherapy based on peptide-drug conjugation-related nanocombinations is promising to achieve highly effective antitumor effects and be used in clinical applications [201].

PA/fluorescence probes can guide the surgical resection of breast cancer and the evaluation of intraoperative margin, with high resolution and specificity [202]. In addition, Dai et al. [203] developed CD44 and scavenger receptor class B1 dual targeting PA/fluorescence probes to guide the precise identification and removal of metastatic SLNs during breast cancer surgery. In addition to guiding intraoperative resection, the probe developed by Wu et al. [204] can be used to monitor tumor senescence in breast cancer models by targeting β-galactosidase (β-gal). Fan et al., based on the caspase-1 nanoreporter (MCNR) of MOFs, noninvasively traced the immune activation process of TAMs and achieved precisely controlled release of TLR7/8 agonists by PA/fluorescence dual-modality imaging. MCNR can enhance T-cell infiltration in breast cancer and other tumor tissues, inhibit tumor growth, and achieve real-time monitoring through caspase-1-mediated specific enzyme digestion [205]. These diverse properties reflect the wide application and innovative breakthrough of PA/fluorescence dual-modality probes in the diagnosis and treatment of breast cancer.

Extensive research and exploration have been conducted on the application of PA and fluorescence dual-modality probes in the precision diagnosis and treatment of breast cancer. These probes have demonstrated significant potential, offering innovative advancements in various aspects of breast cancer management. Their ability to combine the complementary advantages of both PA and FLI modalities has contributed to improved diagnostic accuracy, enhanced treatment monitoring, and better therapeutic outcomes, marking a notable breakthrough in the field of breast cancer precision medicine.

However, the unique characteristics of breast cancer present challenges for PA/fluorescence probe imaging. The heterogeneity of breast cancer, particularly its diverse molecular subtypes, results in unstable target expression. Breast cancer encompasses multiple molecular subtypes—including HER2-positive, triple-negative, and luminal A/B types—each with distinct receptor expression profiles, metabolic states, and TMEs. This diversity leads to variability in biomarker availability (e.g. HER2, EGFR, or estrogen receptor), making it difficult to develop a one-size-fits-all probe [206]. Intratumoral heterogeneity within a single tumor mass can also lead to uneven probe distribution and inconsistent signal intensity during imaging. Furthermore, the high interstitial pressure within breast tumors creates a physical barrier, necessitating probes with enhanced penetration capabilities. Additionally, the distinct metabolic characteristics and unique lymphatic drainage patterns of breast cancer impose stricter demands on the pharmacokinetic properties of the probes. Addressing these challenges is a pressing priority for researchers.

#### Other cancers

In the precise diagnosis and treatment of other cancers, PA/fluorescence dual-modality probes also reflect its excellent performance and development prospects.

##### Cervical cancer

Chen et al. validated their PA/fluorescence dual-modality probe rNGO-PEG/ICG in a mouse model constructed from cervical cancer cells, showing good stability, long blood circulation time, and excellent targeting ability. This probe is expected to be a candidate for further translational studies in early diagnosis and image-guided therapy [207]. Gao et al. [208] substantially increased the signal intensity of probe NIRF and PA by precisely targeting alkaline phosphatase in cervical cancer cells. Similarly, real-time detection of endogenous cysteine (Cys) by PA/fluorescence dual-mode imaging provides a new method for the diagnosis of cervical cancer [209]. Some novel materials, such as carbon defects enriched in boron carbide nanomaterial, exhibit excellent photothermal effects and can be used for tumor phototherapy and synchronous photoacoustic imaging [210].

##### Head and neck cancer

In head and neck cancer, Shakiba et al. validated the delineation of PA/fluorescence by performing real-time intraoperative detection of metastatic lymph nodes using J-aggregates and visual resection [211]. PA/FLI-guided PTT can be achieved by using melanin-dye nanoagent to eliminate laryngeal cancer without obvious side effects [212]. Zheng et al. further integrated PTT, gas therapy (GT) with PA/NIR-II fluorescence. *In situ* released H_2_S can be used not only for gas therapy but also to enhance tumor-specific aggregation of probes, providing promising TME-mediated sustained response strategies [213]. Saad et al. constructed a dual-function antibody conjugate (DFAC) for surgical resection of head and neck cancer and eliminated microscopic lesions through Photoimmunotherapy (PIT), which greatly played the application value of PA/fluorescence dual-modality probe [214].

##### Hepatocellular carcinoma

The PA/fluorescence dual-modality probe targeting Cys can also monitor the occurrence of liver cancer *in vivo* [215]. A dual-modality probe (Au@liposome-ICG) developed by Guan et al. showed good accuracy in Hepatocellular Carcinoma (HCC) detection and resection [216]. The probe developed by Yuan et al. not only achieved signal enhancement of PA and fluorescence but also produced abundant ROS for cancer radiotherapy under X-ray irradiation, realizing the integration of diagnosis and treatment [217].

##### Glioblastoma

Accurate diagnostics plays a crucial role in ensuring optimal treatment outcomes for patients with brain tumors. Sheng et al. reported a PA/fluorescence dual-modality probe with aggregation induced emission (AIE) properties that had better penetration than single-modality probes for accurate brain cancer diagnosis [218]. Lyu et al.’s probe IR-32p also demonstrated high targeting specificity, high tumor contrast, and superior imaging depth in an *in situ* glioblastoma model [219]. PA/fluorescence dual-modality probe can be used for noninvasive mapping of deep microscopic brain tumors. It has irreplaceable advantages in temporal and spatial resolution, signal-to-noise ratio, and imaging depth [220].

##### Lung cancer

The PA/fluorescence dual-modality probe MB-NBD developed by Zhang et al. was used to detect biothiols and was successfully applied to the diagnosis of lung cancer cell models [221]. Long et al. creatively designed a theranostic thermosensitive liposome (PLDD) to exhibit excellent NIR-II fluorescence and PA dual-modality imaging. In addition, the probes contained a stimulatory thermal agent (DTTB) and an immune potentiator STING pathway agonist (DMXAA). It promotes tumor immunity and induces tumor cell death, and has great development potential [222]. However, the high heterogeneity of lung cancer and the tumor’s location present significant challenges for imaging. Developing probes with enhanced metabolic activity and identifying more universal biomarkers offer potential solutions.

##### Pancreatic cancer

Hu et al. recently developed a novel Forster resonance energy transfer (FRET)-mediated PA/fluorescence dual-modality probe, which enables rapid visualization and fluorescence-guided precise surgical resection of pancreatic cancer [223]. In addition, PA/fluorescence probes can be used to monitor ferroptosis in pancreatic cancer to further promote the research and treatment of pancreatic cancer [224].

##### Bladder cancer

A preclinical study of bladder cancer has shown that the use of phage display-derived peptide-based PA/fluorescence dual-modality probes can achieve the detection and guided resection of bladder cancer, greatly reducing the recurrence rate [225].

##### Colorectal cancer

Bi et al. developed endogenous H_2_S-triggered intelligent nanoprobes to further improve the sensitivity and reliability of colorectal cancer diagnosis. The research introduces a novel NIR-II/PA dual-modal imaging approach for the noninvasive and intelligent detection of colorectal cancer [226]. The probes for colorectal cancer need to take into account the significant molecular characteristic differences between the left and right halves of the colon, which is a difficult point that needs to be overcome.

##### Esophageal adenocarcinoma

The probe developed by Chen et al. provides complementary visualization of tumor size in planar and sagittal views, which facilitates the diagnosis and staging of esophageal adenocarcinomas [227].

##### Melanoma

*In vitro*, *in vivo*, and *ex vivo* FLI studies demonstrated that cRGD-conjugated BSQ (BSQ-RGD2) effectively targeted and stained α_v_β_3_ integrin-overexpressing melanoma cells and xenografts, which were clearly visualized by FLI and photoacoustic imaging (PAI). Thus, BSQ-RGD2 is suitable for dual-modality imaging of specific biomarkers in melanoma [228].

##### Thyroid cancer

The probe Affibody-DAP developed by Cheng et al. [229] was able to selectively target EGFR in the FTC-133 subcutaneous mouse model with relatively high photoacoustic and fluorescent signals.

##### Neuroendocrine neoplasms

Xu et al. [230] exploited the complementary properties of PA and FLI to develop a dual-modality probe that significantly improved the detection and intraoperative resection accuracy of neuroendocrine tumors and SLNs.

##### Osteosarcoma

The overall survival rate of patients with OS is not optimistic [231]. In order to improve the treatment and prognosis of OS, Yuan et al. [232] designed and prepared SPN-PT nanoparticles to achieve efficient NIR-II fluorescence/NIR-I PA dual-modality imaging and enhance the therapeutic effect of PTT and PDT effects in OS.

**Figure 5.**
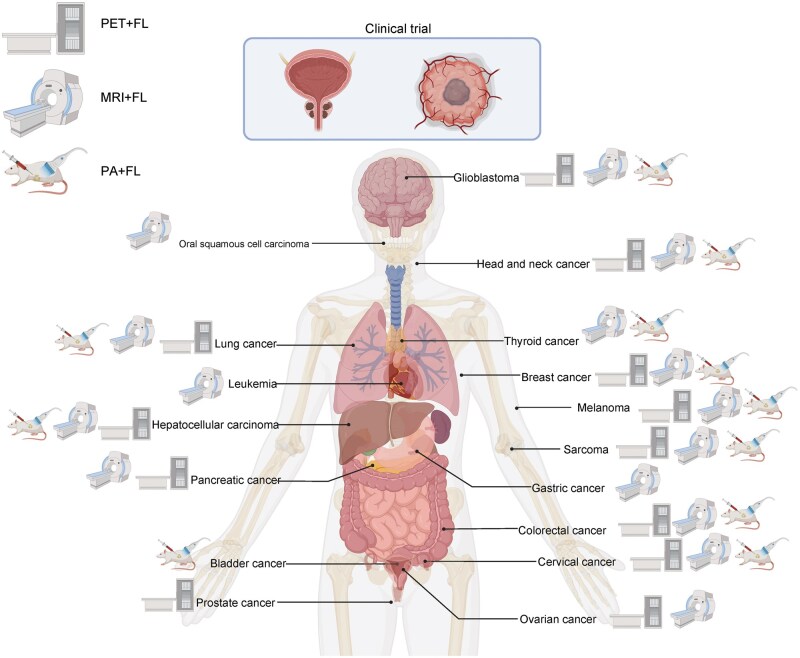
**Applications of dual-modality probes in various cancers.** Breast cancer and glioma demonstrate the most extensive research on three types of dual-modality probes. PET/fluorescence dual-modality probes have undergone human trials in prostate cancer and glioma. FL, fluorescence imaging; PA, photoacoustic imaging. This figure was created with BioRender.

There is no doubt that bimodal probes represented by PA/fluorescence probes have achieved extensive research and application in a variety of cancers (Fig. 5). By combining the complementary properties of PA and FLI, scientists have greatly improved the accuracy and imaging capability of tumor diagnosis and guided resection. Due to the inherent properties of PA/FLI materials, many probes can conveniently combine imaging with various treatment modalities such as PTT, PDT, and so on. This has greatly promoted the integration of precision cancer diagnosis and treatment, which is conducive to improving the prognosis and survival of patient.

## Other combinations of dual-modality molecular probes

In addition to the dual-modality probes introduced above, there are also probes with other combinations of imaging modalities, which also have great application value in the precise diagnosis and treatment of cancers.

### MRI/photoacoustic

The combination of MRI and PA imaging has garnered significant attention due to the complementary strengths of these modalities. MRI/PA dual-modality probes represent a cutting-edge toolset that leverages the high spatial resolution and soft tissue contrast of MRI with the functional imaging capabilities of PA. This synergy enables a more comprehensive understanding of tumor biology, facilitating early detection, accurate staging, and personalized therapeutic interventions [233].

Magnetic resonance imaging/PA dual-modality imaging is often combined with a variety of treatment modalities [234]. Wang et al. [235] designed a GSH-triggered theranostic agent for PA and MR dual imaging-guided photothermal enhanced CDT. A novel nanohybrid, silver-iron oxide nanoparticle designed by Moonshi et al. [236] combines MRI/PA imaging with PTT and shows excellent application potential. Zhang et al. conjugated Janus-type γ-Fe_2_O_3_/SiO_2_ nanoparticles with glucose oxidase to perform PA/T_2_ MR dual-modality imaging of tumors. In addition, the probe could be subjected to cancer starvation treatment and chemodynamic treatment at the same time, which significantly inhibited the growth of breast cancer [237].

Photoacoustic/fluorescence dual-modality imaging has irreplaceable advantages in the diagnosis and treatment of spinal tumors. Qian et al. [238] improved the efficiency of spinal cancer elimination by synergistic chemotherapy/CDT, and realized the visualization of tumor treatment and material degradation *in vivo* using MR and photoacoustic dual-modality imaging. In addition, dual-modality MRI/PA probes such as TD-FeCl_3_ not only have excellent imaging capability but also act as natural ferroptosis tracers to deplete GSH and further enhance tumor ferroptosis [239].

Magnetic resonance imaging/photoacoustic dual-modality probes can provide comprehensive, multiparametric imaging data, coupled with their potential for real-time monitoring and targeted therapy, makes them invaluable for advancing tumor diagnosis and treatment. As research progresses, the development of advanced probes with improved biocompatibility and targeting specificity is expected to broaden clinical applications, enhancing patient outcomes in cancer treatment.

### Single-photon emission computed tomography/fluorescence

Single-photon emission computed tomography (SPECT) uses gamma rays emitted from a radiopharmaceutical tracer to create 3D images of internal organs or tissues, based on the distribution of the tracer in the body. Its good penetration depth and high spatial resolution of FLI achieve a good complement, reflecting the great advantages of dual-modality in the precise diagnosis and treatment of tumors [240, 241].

Derks et al. [242] developed a dual-modality probe targeting PSMA for guided resection of PCa combined with PDT. Zhou et al. [243] developed the probe ^99m^Tc-ICG-HSA nanoparticles for noninvasive SLN identification and biopsy guidance by SPECT/FLI. The dual-modality probe constructed by Deng et al. [244] showed excellent performance for SLN localization by SPECT imaging and NIRF for intraoperative real-time monitoring and SLN PTT. Although the sensitivity of SPECT imaging is not as high as that of PET, it still holds significant advantages and application value in areas such as SLN localization, bone scanning, and beyond.

### PET/photoacoustic

The combination of PET and photoacoustic imaging improves the sensitivity of imaging and achieves multidimensional information complementation. Li et al. obtained multifunctional nanoparticles by labeling the BDT-DPP polymer with [^64^Cu]. After injection into HepG2 tumor model, the probes showed excellent PA and PET imaging capabilities [245]. Yang et al. [246] constructed a series of [^64^Cu]-labeled perylene diimide nanoparticles of different sizes for PET/PA dual-modality imaging and PTT of lymph nodes and tumors. Due to the difficulty of integration and application requirements, the development and research of PET/PA dual-modality probes are limited. Optimizing the probe structure and utilizing its characteristics to broaden its application value in tumor diagnosis and treatment are the key issues to tap the potential of PET/PA dual-mode probes in the next step.

### Magnetic particle imaging/fluorescence

Magnetic particle imaging (MPI) combined with FLI represents a promising dual-modal imaging strategy for tumor diagnosis and therapy. Philips Research first introduced the MPI imaging in 2005 [247]. It utilizes superparamagnetic nanoparticles (SPIONs) as tracers to provide high spatial and temporal resolution without ionizing radiation and enables 3D real-time imaging [248, 249]. When coupled with FLI, this dual-mode probe benefits from the high sensitivity and molecular specificity of fluorescence, allowing for precise tracking of biomolecular interactions at the cellular or subcellular level. Wang et al. [250] used MPI/fluorescence dual-modality probes to detect metastatic lymph nodes in breast cancer and achieving promising results. Li et al. [251] performed *in vivo* evaluation and tracking of tumor-infiltrating lymphocytes using an MPI/FLI system, providing a novel method for personalized immunotherapy. Additionally, MPI imaging can be well combined with PTT, which provides a promising direction for the development of dual-modality probes in the future [252]. Although MPI imaging offers significant advantages, such as the absence of depth limitations, the development of MPI/fluorescence dual-modality probes remains in its early stages, with further improvements needed in targeting and long-term safety. Continued optimization of the probe structure, along with further experimental validation, is necessary for future progress.

## Challenges and future prospects of dual-modality molecular probes

Dual-modality molecular probes have emerged as a powerful tool in the precision diagnosis and treatment of tumors, offering significant advantages over single-modality probes by integrating complementary imaging techniques to provide a more comprehensive understanding of tumor biology. Unlike single-modality probes, which are limited by the inherent constraints of their respective imaging techniques, dual-modality probes combine the strengths of two modalities to overcome these limitations. For example, PET/fluorescence probes merge the high sensitivity and quantitative capabilities of PET with the high spatial resolution and real-time imaging of fluorescence, enabling both deep-tissue imaging and precise intraoperative guidance. Similarly, MRI/fluorescence probes combine the exceptional soft-tissue contrast and anatomical detail of MRI with the advantages of FLI, providing a more complete picture of tumor morphology and molecular characteristics. These synergistic advantages make dual-modality probes uniquely suited for addressing the complex challenges of tumor diagnosis and treatment.

Despite these advancements, several critical challenges must be addressed to fully realize the clinical potential of dual-modality probes and facilitate their widespread adoption. A primary concern is the biocompatibility and potential toxicity of these probes. Many incorporate heavy metals, radionuclides, or synthetic nanomaterials, which may provoke immune responses or cause adverse *in vivo* effects. For instance, the use of Gd contrast agents in MRI/fluorescence probes poses the risk of Gd deposition, potentially leading to chronic damage, particularly in regions like the brain [253]. Therefore, iron-based dual-mode probes are more likely to facilitate the progression of MRI/fluorescence dual-modal imaging into clinical trial stages, as they can employ components analogous to iron supplements, ensuring enhanced safety. Moreover, the long-term effects of nanoparticles used in dual-modality probes remain inadequately understood [254]. Ensuring the biological safety of these probes necessitates comprehensive toxicity testing, the development of biodegradable materials, and the exploration of alternative, less toxic components.

The design and production of dual-modality probes often involve complex, multistep chemical processes that are both time-consuming and technically challenging. The need to integrate two distinct imaging modalities without compromising their individual functionalities adds to the synthesis complexity. For example, developing activatable imaging probes capable of simultaneously amplifying both fluorescence and PA signals presents a significant challenge, as the mechanisms that enhance light absorption for PA imaging often result in the quenching of fluorescence signals [255]. Furthermore, achieving batch-to-batch consistency and scalability remains a major hurdle, particularly for probes intended for clinical use. Variability in synthetic protocols, raw material quality, and reaction conditions can lead to inconsistent probe behavior, posing risks in safety and efficacy. Addressing this issue requires the development of standardized, validated manufacturing processes and implementation of stringent quality control measures, especially when scaling up for clinical-grade production. Currently, the cost of developing and synthesizing dual-modality probes is relatively high, which hinders their widespread adoption in clinical practice. Therefore, optimizing probe structure design and developing automated synthesis platforms will help streamline the technical process, reduce costs, and improve probe performance.

While dual-modality probes are designed to selectively target tumor-specific biomarkers, achieving high specificity *in vivo* remains a significant challenge. Nonspecific uptake in healthy tissues, off-target binding, and interactions with the reticuloendothelial system can result in false-positive signals and reduced diagnostic accuracy. Additionally, the metabolic stability and clearance of dual-modality probes are crucial factors affecting both their performance and safety. To enhance the metabolic stability of these probes, scientists often incorporate molecules such as PEG [44, 107]. Further studies are needed to improve the targeting specificity and metabolic stability of the dual-modality probes.

Despite these challenges, the future of dual-modality molecular probes appears exceptionally promising. Multiomics approaches are emerging as powerful tools to address the issue of target instability. Proteomics enables the dynamic monitoring of protein expression and posttranslational modifications in response to the TME or treatment interventions, thereby helping to validate robust and temporally relevant biomarkers for probe targeting [256]. By integrating multiomics data, we are able to identify subtype-specific or progression-dependent molecular signatures, facilitating the development of personalized probes that remain effective despite tumor heterogeneity and biomarker fluctuations to overcome target instability.

The application of artificial intelligence (AI) and machine learning (ML) to dual-modality imaging probes has opened new avenues for integrating and interpreting the increasingly complex data generated by modern imaging technologies [257, 258]. AI and ML can enhance image quality, automate organ segmentation, and facilitate the extraction of subtle imaging features that reflect underlying tumor biology [259]. These tools enable more accurate stratification of patients and early prediction of treatment response by correlating imaging features with biological and therapeutic outcomes [12]. Recent developments also point to the potential of integrating *in vivo* imaging data with *ex vivo* multiomics or liquid biopsy results, forming a comprehensive diagnostic framework [260]. As total-body PET systems and other high-throughput imaging modalities continue to evolve, AI-driven data integration will be central to managing the complexity of multidimensional information and advancing the clinical translation of dual-modality molecular probes in personalized oncology.

**Figure 6.**
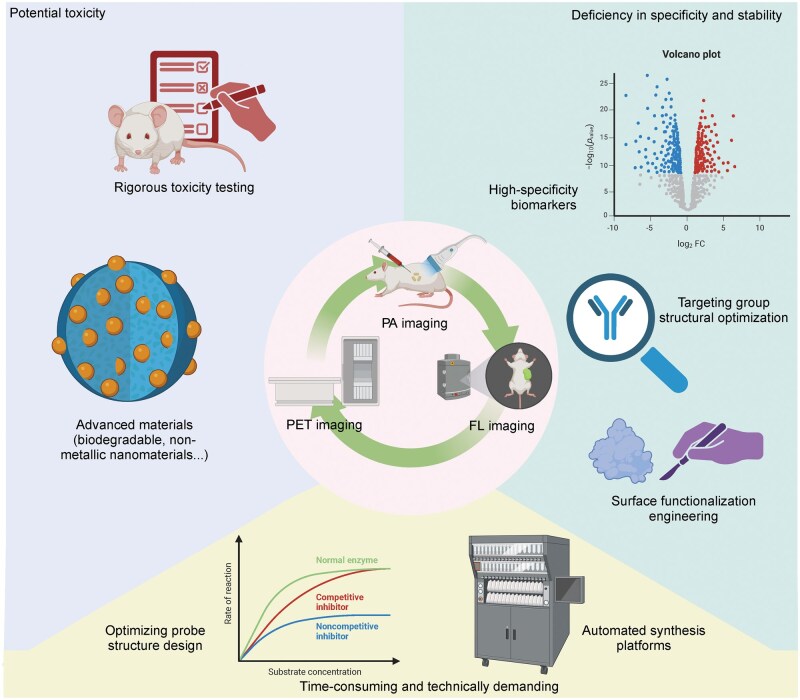
**Challenges and potential solutions for dual-modality probes in tumor diagnosis and treatment. **Currently, dual-modality probes face three major challenges in clinical applications: potential toxicity, insufficient stability and specificity, and immature large-scale production. To address toxicity concerns, rigorous toxicity evaluations combined with advanced biocompatible materials could mitigate biological risks. Improving stability and specificity requires a dual approach: leveraging multiomics technologies to identify high-precision molecular targets, and structurally optimizing probes through strategies like refining targeting groups and enhancing linker stability. For scalable manufacturing, establishing standardized production protocols and integrating automation systems with AI-driven quality control is critical to ensure batch consistency and cost efficiency. These integrated strategies not only address technical bottlenecks but also accelerate the translational potential of dual-modality probes in precision oncology. This figure was created with BioRender.

In terms of imaging modality exploration, the introduction of novel imaging modalities such as surface-enhanced Raman scattering, which leverages bioorthogonal chemical reactions, overcomes the sensitivity limitations of traditional imaging techniques, and expands potential options for dual-modal imaging [261, 262]. Additionally, the development of theranostic probes that integrate diagnostic and therapeutic capabilities offers significant potential for personalized medicine, enabling clinicians to monitor treatment responses in real-time and adjust therapies accordingly. However, there remains a shortage of sufficient clinical trials to validate the efficacy of dual-modality probes. Many existing studies are limited by small sample sizes, single-center designs, or short-term outcome assessments, which reduce statistical power and limit the generalizability of the results. Large-scale, multicenter clinical trials with robust methodology are urgently needed to establish the clinical utility and long-term safety profiles of these agents. With further exploration and optimization, it is anticipated that these probes will more effectively combine diagnosis, surgical guidance, and various treatment modalities, thereby realizing their full clinical application potential (Fig. 6).

## Conclusion

In conclusion, dual-modality molecular probes represent a substantial advancement over single-modality probes, providing unparalleled capabilities for the precise diagnosis and treatment of tumors. Specifically, these probes offer significant advantages and considerable potential in tumor detection, intraoperative guidance for resection, and the integration of multiple therapeutic approaches.

However, overcoming challenges such as biosafety, synthesis complexity, and targeting specificity is essential for the successful translation of dual-modality probes into clinical practice. Through the advancement of technology and fostering interdisciplinary collaboration, these limitations can be addressed, unlocking the full potential of these probes. The future of this field lies in the development of robust, targeted, and cost-effective probes that can seamlessly integrate into clinical workflows, ultimately enhancing patient outcomes and advancing the frontiers of precision oncology.
